# Developing a readiness evaluation framework for public hospital-led digital home nursing services in China: a Delphi-AHP study

**DOI:** 10.1186/s12912-026-05311-6

**Published:** 2026-08-29

**Authors:** Yuzhe Wang, Zimin Wang, Yingchun Liu, Guiming Yan, Fang Hu, Xiaodan Wu, Tianzhi Yu

**Affiliations:** 1 School of Management, Tianjin University of Traditional Chinese Medicine, Tianjin, China; 2 The First School of Clinical Medical, Tianjin Medical University, Tianjin, China; 3 School of Nursing, Tianjin Medical University, Tianjin, China; 4 Internet Hospital, Tianjin Medical University General Hospital, No. 154 Anshan Rd, Heping District, Tianjin, 300052 China; 5 School of Economics and Management, Hebei University of Technology, Tianjin, China; 6 Hospital Administration Office, Tianjin Medical University General Hospital, Tianjin, China

**Keywords:** Internet plus nursing services, Digital home nursing, Readiness assessment, Evaluation framework, Delphi-AHP

## Abstract

**Background:**

Internet Plus Nursing Services have become an important public hospital-led model for extending professional nursing care from hospitals to patients’ homes through digital platforms in China. Although previous studies have examined service quality, patient experience, nurse participation, and platform functions, little attention has been paid to whether public hospitals possess the system readiness required to deliver safe, sustainable, traceable, and accountable digital home nursing. This study aimed to develop a readiness evaluation framework for public hospital-led digital home nursing services and determine the relative priority of its indicators.

**Methods:**

Porter’s Five Forces model was contextualized as an organizing framework for readiness domains after consideration of established health-system and implementation perspectives. An initial indicator pool was developed through literature review, policy document analysis, and preliminary expert consultation. Two rounds of Delphi consultation were conducted to refine the second- and third-level indicators. A score-informed adaptation of the analytic hierarchy process (AHP) was subsequently used to estimate indicator weights from the second-round Delphi importance scores. Expert authority, consensus, judgment-matrix consistency, and the sensitivity of the resulting priorities to small changes in the score-to-Saaty conversion boundaries were evaluated.

**Results:**

52 experts participated in the first Delphi round, and 48 completed the second round. The expert authority coefficient was 0.74. Kendall’s W increased from 0.150 to 0.196 for the second-level indicators and from 0.144 to 0.205 for the third-level indicators, with all tests statistically significant (*P* < 0.001). The final framework comprised five first-level domains, 14 second-level indicators, and 43 third-level indicators. Workforce and Digital Platform Infrastructure received the highest first-level weight (32.29%), followed by Patient Adoption, Payment, and Value Recognition (24.47%) and Operational Quality, Safety, and Accountability (18.54%). All applicable AHP judgment matrices met the consistency criterion, with CR values ranging from 0.0088 to 0.0516. Sensitivity analyses showed that small shifts in the conversion boundaries did not materially alter the overall priority structure.

**Conclusions:**

This study developed a structured readiness evaluation framework for public hospital-led digital home nursing services in China. The findings highlight workforce and digital platform infrastructure, patient adoption and payment support, and operational quality, safety, and accountability as priority readiness conditions. The framework provides a structured reference for assessing the conditions required for safe, traceable, and sustainable digital home nursing, while further validation using multicenter and real-world service data is warranted.

**Supplementary Information:**

The online version contains supplementary material available at https://doi.org/10.1186/s12912-026-05311-6.

## Introduction

Digital health technologies are increasingly extending professional nursing care from institutional settings to patients’ homes and reshaping nursing delivery as a platform-supported medical service system. In response to population aging, chronic disease burden, and growing demand for postdischarge and home-based care, digital platforms have become an important infrastructure for organizing nursing services beyond hospital walls. This shift can improve continuity of care and service accessibility, but it also makes nursing practice more complex because nurses must deliver professional care in less controlled home environments and rely on digital platforms for service coordination and organizational support. In China, this service transformation has taken the form of “Internet Plus Nursing Services,” a policy-supported model of digital home nursing in which registered nurses from medical institutions provide offline home visits through internet-based platforms [1]. Since the National Health Commission launched the pilot program in 2019, this model has gradually expanded from postdischarge continuing care to specialized nursing, chronic disease management, geriatric nursing, rehabilitation guidance, and other home-based nursing services [2].

Driven by policy support, technological advancement, and increasing demand for home-based nursing care, Internet Plus Nursing Services have expanded rapidly in China. By the end of 2022, more than 2000 medical institutions had provided these services, covering more than 60 home-based medical nursing items in 7 categories [3]. However, the expansion of this model should not be understood only as an increase in service volume. More importantly, it has created a platform-mediated digital nursing system in which public hospitals must respond to multiple system pressures, including service entry requirements, nursing workforce supply, platform resource dependence, patient payment and choice, substitute forms of home-based care, and provider-level quality and accountability. In this environment, primary health care institutions, private medical institutions, third-party platforms, community services, elderly care institutions, and informal caregivers may meet different levels of home-based care needs, but their roles also make it necessary to clarify the boundary between professional nursing services and substitute care. For public hospital-led digital nursing platforms, the central challenge is therefore not merely how to expand Internet Plus Nursing Services, but how to organize qualified nurses, digital platforms, payment arrangements, quality control, risk management, and responsibility tracing into a safe, traceable, and accountable home-based nursing system [4, 5].

Existing research on Internet Plus Nursing Services has examined patient demand, nurses’willingness to participate, service quality, nursing competency, and platform functions [6–15]. Studies have shown that patients requiring postdischarge care, chronic disease management, and home-based nursing support are important target users of Internet Plus Nursing Services, emphasizing the importance of accessibility, affordability, continuity of care, and clear service scope [6, 7]. Other studies have found that nurses’ participation is influenced by income, service distance, occupational risk, training support, platform support, perceived professional benefits, and safety-related support [8–11]. In addition, studies on service capability indicators, nursing competencies, mobile applications, and nurses’ service experiences have highlighted standardized service processes, specialized nursing skills, platform functionality, information quality, privacy protection, system stability, and security assurance [12–15]. However, these findings remain fragmented and have not been integrated into a structured framework for assessing whether public hospital-led digital nursing platforms have the system readiness to coordinate workforce protection, platform support, payment arrangements, service boundaries, quality monitoring, and accountability in home-based care.

Internationally, related models such as telehealth nursing, hospital-at-home programs, telenursing, virtual nursing, postdischarge nursing, and digitally coordinated home care have similarly expanded professional care beyond traditional clinical settings [16–20]. Although these models differ in service organization, licensure arrangements, and payment mechanisms, they reveal a common feature of digital home-based care: professional services must be organized within a multi-actor system involving health care institutions, nurses, patients, digital platforms, payers, community resources, and alternative care providers. This system creates several interrelated pressures, including who is qualified to provide home-based professional care, whether sufficient nursing and technological resources are available, whether patients can afford and trust digital services, how professional nursing is distinguished from substitute forms of care, and whether providers can ensure quality, safety, responsiveness, and accountability across institutional and home settings. These shared pressures suggest that digital home nursing requires a structured framework that can organize access, resource, patient, substitute-care, and provider-level accountability issues into an assessment of system readiness.

Given these interrelated pressures, a structured framework is needed to organize the readiness conditions that shape public hospital-led digital home nursing. Porter’s Five Forces model provides a useful analytical structure because it distinguishes five types of forces within a multi-actor service environment: service entry conditions, resource dependence, user choice, substitute services, and provider-level performance [21]. Recent health care research has also applied this model to home-based care; for example, Baugh et al. used Porter’s Five Forces to develop a conceptual framework for acute home-based care for patients with cancer, showing how entry conditions, resource relationships, patient needs, substitute services, and provider pressures may influence the development of home-based care models [22]. In this study, the model was used as an organizing framework and was contextualized as a regulatory and health information system readiness lens for Internet Plus Nursing Services. Under this interpretation, new entrants reflect service access requirements, institutional qualifications, nurse practice conditions, and platform construction barriers; suppliers reflect nursing workforce supply, professional risk protection, platform support, and technological resource dependence; buyers reflect patient payment capacity, service choice, trust, and perceived value; substitutes reflect the boundary between registered nursing services and alternative forms of home-based care; and industry rivalry reflects provider-level differences in service quality, responsiveness, safety assurance, platform-supported documentation, and accountability tracing. This contextualization retains the structural logic of Porter’s model while reframing the five forces as readiness domains for assessing whether public hospital-led digital nursing platforms can organize safe, traceable, and accountable home-based care through appropriate service access, resource support, patient adoption, service boundary management, quality monitoring, and accountability tracing.

Therefore, this study aimed to develop a regulatory and health information system readiness framework for public hospital-led digital home nursing services in China, using Internet Plus Nursing Services as the empirical context. By integrating literature review, policy document analysis, Delphi expert consultation, and analytic hierarchy process weighting, this study sought to identify the key readiness domains required for safe, traceable, and accountable platform-mediated nursing care. Rather than evaluating Internet Plus Nursing Services only as a service expansion model, this study focuses on whether public hospital-led digital nursing platforms are prepared to coordinate access requirements, workforce and platform resources, patient payment and value recognition, substitute care boundaries, quality monitoring, and accountability tracing. The proposed framework may help public hospitals assess weaknesses in digital home nursing system design and may provide regulators with a structured reference for improving platform governance, service standards, patient safety, and organizational accountability.

## Methods

### Study design and readiness framework development

This methodological study combined evidence synthesis, expert consensus, and quantitative priority weighting to develop a regulatory and health information system readiness framework for public hospital-led digital home nursing services in China [23, 24]. Internet Plus Nursing Services were used as the empirical context because they represent a policy-supported and platform-mediated model in which registered nurses from medical institutions provide home-based nursing care through internet-based platforms.

In selecting the conceptual framework, we considered established health-system and implementation frameworks that could potentially organize readiness for digital home nursing. The WHO health-system building blocks provide a system-level structure covering service delivery, health workforce, health information systems, financing, governance, and access to essential technologies, and are particularly useful for assessing core health-system functions and capacities [25]. CFIR is primarily designed to identify determinants that influence the implementation of innovations across the innovation, inner setting, outer setting, individuals, and implementation process [26]. Similarly, the NASSS framework focuses on the adoption, abandonment, scale-up, spread, and sustainability of health technologies by examining complexity across the technology, adopter system, organization, wider context, and their interactions over time [27, 28]. These frameworks therefore provide important perspectives on health-system capacity and implementation processes, but they were less directly aligned with the specific purpose of this study: to organize readiness conditions arising from the relationships among hospitals, nursing and platform resources, patients, alternative care providers, and other service actors within a regulated digital home-care environment.

Porter’s Five Forces model was therefore selected as the organizing framework because its relational structure explicitly distinguishes entry conditions, dependence on key resource providers, service-user choice and payment, substitute forms of care, and provider-level pressures within a multi-actor service environment [21]. These features corresponded closely to the structural questions facing public hospital-led Internet Plus Nursing Services: which institutions and professionals are eligible to provide the service, whether nursing and digital-platform resources are sufficiently available, whether patients can access and afford the service, how alternative home-care arrangements affect service boundaries and continuity, and whether providers can maintain quality, safety, responsiveness, and accountability. The model was not used to assess commercial competitiveness or profitability. Rather, its five-force structure was contextualized as a readiness lens for identifying the relational and organizational conditions required for safe and sustainable digital home nursing. In this respect, Porter’s model was used to complement, rather than replace, health-system and implementation perspectives by providing a more explicit structure for the interdependencies among service actors that were central to the study objective.

**Fig. 1:**
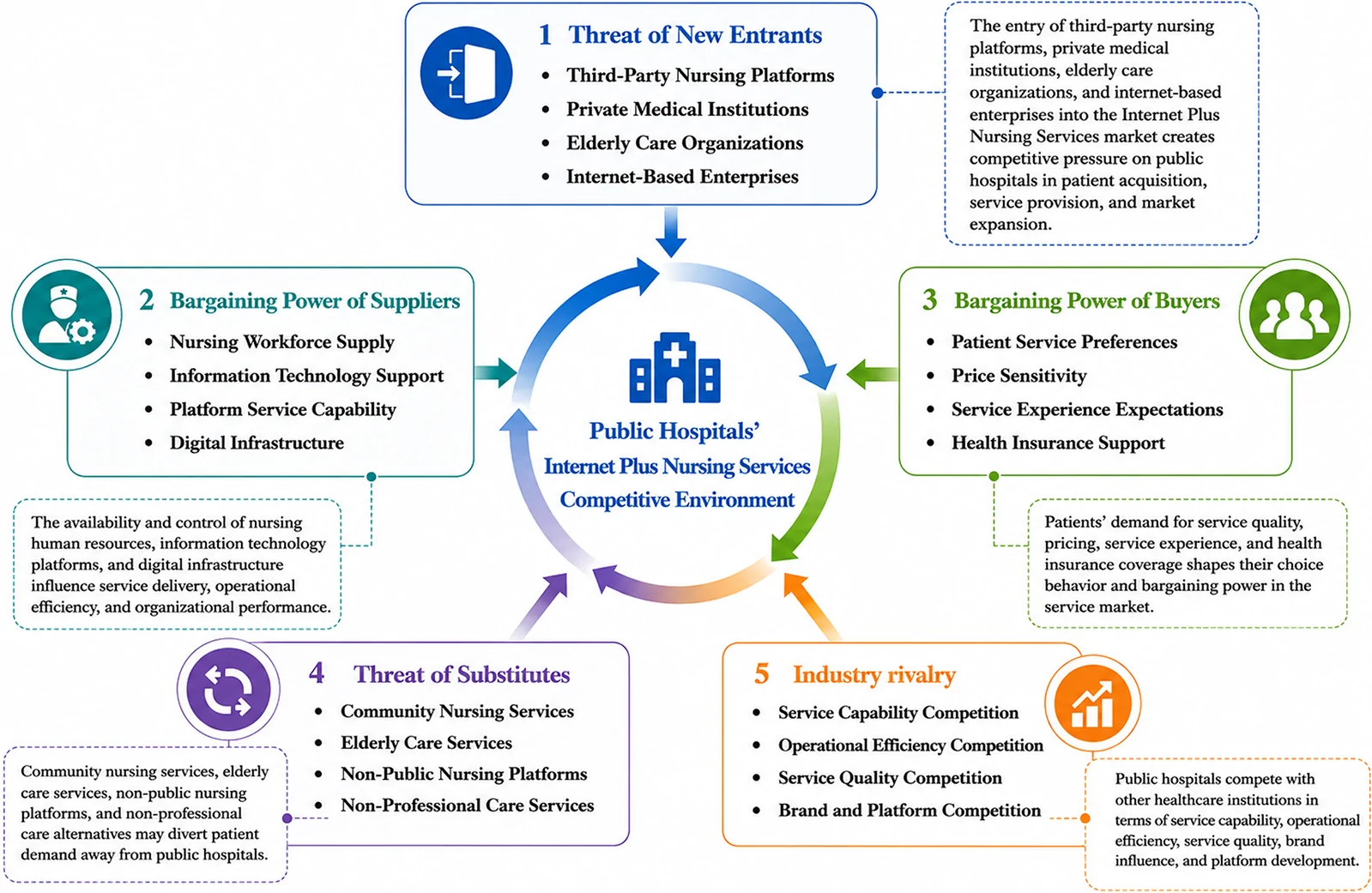
Conceptual framework of regulatory and health information system readiness for digital home nursing services

Based on this framework selection, and before the Delphi consultation, the five forces were contextualized within the Internet Plus Nursing Services setting and used to define the first-level readiness domains of the framework. New entrants were reinterpreted as service entry readiness, including policy access, institutional qualification, nurse practice requirements, and platform construction barriers. Suppliers were reinterpreted as nursing workforce and platform resource readiness. Buyers were reinterpreted as patient adoption and payment readiness. Substitutes were reinterpreted as substitute-care boundary readiness, focusing on the distinction between registered nursing services and alternative forms of home-based care. Industry rivalry was reinterpreted as provider-level operational readiness, including service quality, responsiveness, safety assurance, documentation, and accountability. This contextualization allowed the model to retain its structural logic while aligning it with the regulated, platform-supported, and safety-sensitive nature of digital home nursing.The contextualized conceptual framework is shown in Fig. 1.

**Fig. 2:**
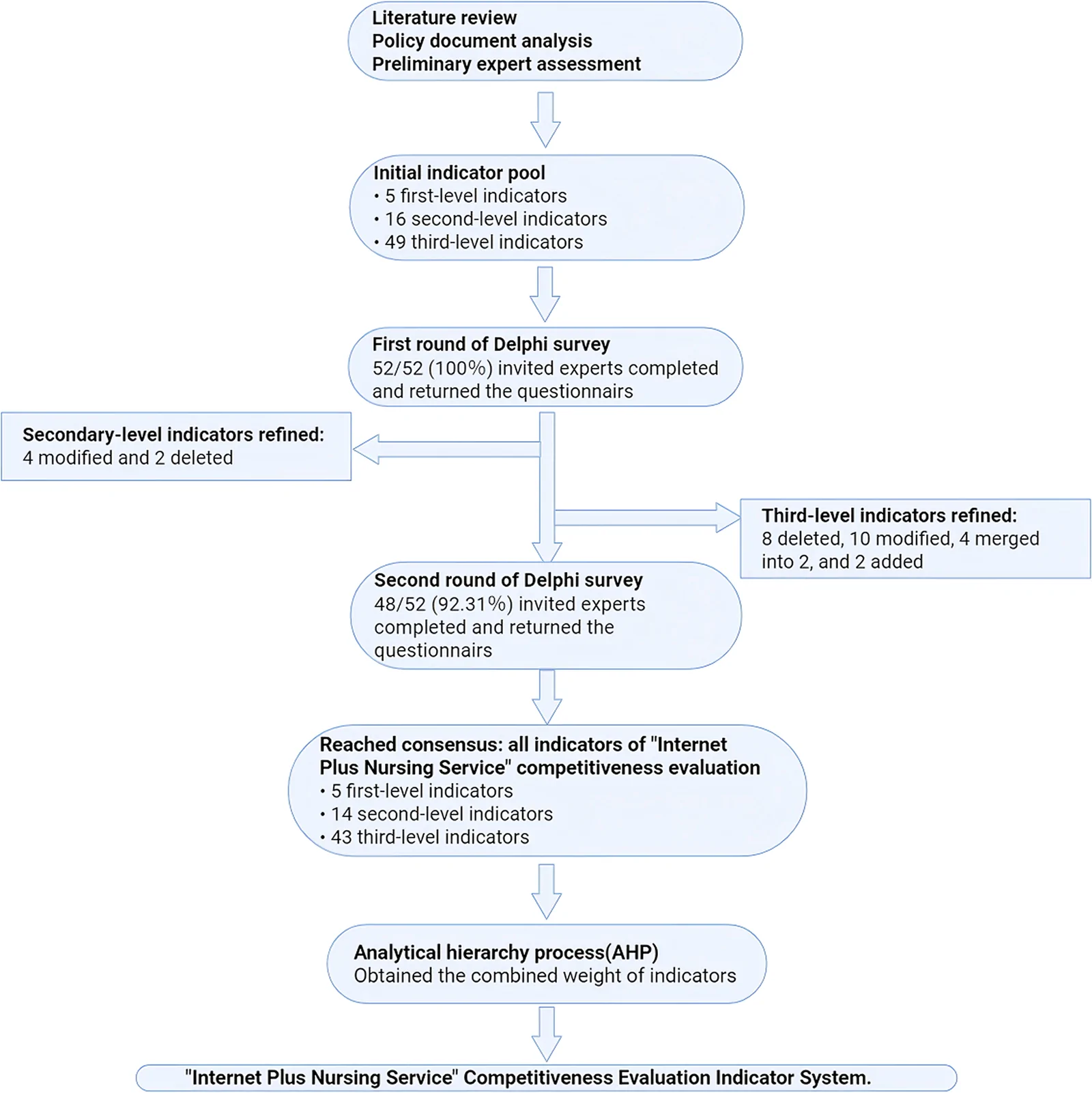
Study workflow for indicator development and priority weighting

The study was conducted in four stages. First, the contextualized Five Forces model was used to construct the conceptual framework and define the first-level readiness domains. Second, an initial indicator pool was developed through literature review, policy document analysis, and preliminary expert consultation. Third, the indicators were screened and revised using a two-round Delphi expert consultation process. Fourth, a Delphi-derived analytic hierarchy process was used to estimate the relative priority weights of indicators at each hierarchical level, and consistency testing was performed to assess the logical consistency of the judgment matrices. The overall research workflow is shown in Fig. 2.

### Development of the initial indicator pool

The initial indicator pool for the regulatory and health information system readiness framework was developed through literature review, policy document analysis, and preliminary expert consultation. The purpose of this stage was to identify candidate indicators that reflected both the evidence base and the policy and operational requirements of public hospital-led Internet Plus Nursing Services. First, a structured literature search was conducted in PubMed, China National Knowledge Infrastructure, and Wanfang Database, with the search period ending on January 1, 2026. The search strategy was organized around five topic modules: Internet Plus Nursing Services and digital nursing, home-based and continuing nursing care, platform-mediated service delivery and health information system readiness, service governance and patient safety, and evaluation indicator development. Terms related to service-system structure and provider-level performance were also included to capture studies using organizational or structural perspectives relevant to the contextualized Five Forces framework. Two researchers independently screened titles, abstracts, and full texts and extracted candidate indicators. Disagreements were resolved through discussion, and unresolved issues were reviewed by a third researcher. After duplicate records were removed, 1288 records were retained for title and abstract screening. Following full-text assessment, 29 studies were finally included for indicator extraction. The search modules, database-specific search strategies, eligibility criteria, and screening and indicator extraction procedures are provided in Supplementary 1 Table S1. Candidate indicators identified from the literature and policy documents were mapped to the five contextualized readiness domains, while relevant content beyond the original business-strategy terminology was retained when it reflected health-system, implementation, regulatory, or digital-care requirements.

Second, to ensure that the indicator system was aligned with the policy environment and practical boundaries of Internet Plus Nursing Services in China, 123 policy documents were reviewed, including 20 national-level documents and 103 local policy documents from Beijing, Tianjin, Shanghai, Jiangsu, Zhejiang, and Guangdong. Policy requirements related to service access, nurse practice, platform construction, medical insurance payment, service pricing, quality and safety, risk prevention and control, and regulatory supervision were extracted.Policy clauses were transformed into candidate indicators when they described institutional requirements, platform functions, service standards, payment arrangements, safety requirements, or accountability mechanisms relevant to public hospital-led digital home nursing. The distribution and complete list of the included national and local policy documents are presented in Supplementary 2 Tables S2–S9.

Finally, two experts in health policy and hospital management were invited to conduct preliminary validation of the candidate indicators. They mainly assessed the theoretical relevance, clarity of wording, practical measurability, and rationality of the hierarchical structure of the indicators. Based on expert feedback, the research team preliminarily revised the indicator names, conceptual meanings, and hierarchical classification. After integrating evidence from the literature, policy documents, and expert opinions, an initial pool was developed for the subsequent Delphi consultation. The initial pool contained 5 first-level readiness domains, 16 s-level indicators, and 49 third-level candidate indicators.

### Expert panel and eligibility criteria

Experts for the Delphi consultation were selected using purposive sampling to ensure multidisciplinary expertise and practical representativeness in public hospital-led digital home nursing services. Because the proposed framework involved nursing practice, platform-mediated service delivery, hospital management, health information system readiness, and regulatory governance, the expert panel was designed to include professionals with experience in clinical nursing services, nursing management, nursing education, internet hospital operation, health information management, and internet-based health policy. This composition was intended to ensure that indicator judgments reflected both professional nursing standards and the operational and governance requirements of platform-mediated home nursing services.

The inclusion criteria were as follows: (1) familiarity with Internet Plus Nursing Services or related digital health service models; (2) professional experience in internet hospital management and operation, clinical nursing, nursing management, nursing education, health information management, or internet-based health policy; (3) at least 5 years of professional experience; (4) an intermediate or higher professional title; and (5) voluntary participation and the ability to complete the Delphi consultation. Experts whose professional background was not relevant to the study topic or who were unable to complete the questionnaire independently were excluded.

### Delphi consultation process

A two-round Delphi consultation was conducted to screen, revise, and finalize the candidate indicators. The Delphi method was used because it allows anonymous expert judgment, structured feedback, and iterative refinement of indicators when direct empirical measurement is limited. The consultation process was designed with reference to reporting recommendations for consensus-based health research [29, 30].

The consultation questionnaire was developed based on the initial indicator pool and included the study objectives, completion instructions, and operational definitions of candidate indicators. The questionnaire consisted of four sections: (1) experts’ basic information; (2) ratings of the importance, feasibility, and applicability of each indicator; (3) experts’ familiarity with the study topic; and (4) the basis for expert judgment. Importance, feasibility, and applicability were rated using a 5-point Likert scale, where 5 indicated “very important,” “highly feasible,” or “highly applicable,” and 1 indicated “not important,” “not feasible,” or “not applicable.” Experts were also invited to provide open-ended comments on whether indicators should be revised, merged, deleted, or added. The first- and second-round Delphi expert consultation questionnaires are provided in Supplementary Material 5 and Supplementary Material 6.

The familiarity coefficient (Cs) was determined by expert self-assessment and classified into five levels: extremely familiar [1.00], very familiar [0.80], moderately familiar [0.60], slightly familiar [0.40], and unfamiliar [0.20]. The judgment coefficient (Ca) was used to quantify the basis of expert opinions, including theoretical analysis, practical experience, literature evidence, and intuitive judgment, with scores assigned according to the degree to which each basis influenced expert judgment [31, 32]. The quantification criteria, standardization procedure, and calculation method for the expert authority coefficient are presented in Supplementary 3 Table S10. The expert authority coefficient (Cr) was calculated as the arithmetic mean of Ca and Cs.

In the first round, experts independently rated the candidate indicators and provided written suggestions. The research team summarized the quantitative results and anonymized qualitative comments, and revised the indicator pool according to the predefined screening criteria and expert feedback. In the second round, experts received the statistical results of the first-round ratings and a summary of anonymized comments, allowing them to reconsider their judgments in relation to the group opinion. The Delphi consultation was terminated after the second round because all retained indicators met the predefined screening criteria, no major structural revisions were proposed, and the coordination of expert opinions reached an acceptable level. The final indicator system was then established for priority weighting.

### Indicator screening criteria

Indicators were screened based on the mean importance score, full-score frequency, coefficient of variation, and experts’ written comments. The mean importance score reflected the overall perceived importance of each indicator, full-score frequency represented the proportion of experts assigning the highest rating, and the coefficient of variation reflected dispersion in expert ratings. Based on previous Delphi-based indicator development studies, indicators with a mean importance score below 3.5, a coefficient of variation greater than 20%, or a full-score frequency below 20% were considered candidates for deletion or modification [32–34]. These thresholds are not universal Delphi standards but were predefined as study-specific operational criteria based on cut-offs commonly used in health care indicator-development studies using 5-point importance ratings. Final decisions were not based on the numerical thresholds alone but also incorporated experts’ written comments, conceptual relevance, clarity, and practical measurability.

Indicator revision was not determined mechanically based only on statistical thresholds. The research team also considered experts’ written comments, the relevance of each indicator to the contextualized readiness framework, the clarity of wording, practical measurability, and the rationality of the hierarchical structure. Indicators with clear conceptual relevance but ambiguous wording were revised; indicators with overlapping meanings were merged; indicators that were insufficiently related to the study topic or framework structure were deleted; and important content suggested by experts but not covered in the initial pool was added. After the first-round results and expert comments were summarized, the revised indicator pool was used to develop the second-round consultation questionnaire.

### Delphi-derived AHP weighting

The analytic hierarchy process (AHP) was used to estimate the relative priority of indicators within the hierarchical readiness framework. In conventional AHP, relative priorities are elicited directly through pairwise comparisons using the Saaty 1–9 scale. In the present study, however, the Delphi questionnaires were designed primarily for indicator screening and consensus formation and therefore collected 5-point importance ratings rather than direct pairwise-comparison judgments. We consequently used a score-informed adaptation of AHP in which the mean importance scores obtained from the second Delphi round were used to construct pairwise judgment matrices. This procedure should therefore be regarded as an indirect weighting approach based on Delphi-derived importance scores rather than a conventional direct-elicitation AHP procedure.

For indicators belonging to the same parent domain, the difference between the second-round mean importance scores of indicators m and n was calculated as I_m_−I_n_. The absolute difference was then converted to the Saaty 1–9 scale according to the predefined intervals presented in Table 1. In AHP, the Saaty scale is used to represent increasing levels of relative importance, with 1 indicating equal importance and reciprocal values indicating the opposite direction of preference [35, 36]. Accordingly, a difference of 0 was assigned a value of 1; positive differences indicated greater relative importance of indicator m, whereas negative differences were represented by the reciprocal of the corresponding scale value.

The conversion intervals were predefined as an operational discretization of differences derived from the 5-point importance scale. Consecutive 0.25-point intervals were used so that larger differences in Delphi importance scores corresponded to progressively stronger levels of relative importance on the Saaty scale. Similar score-difference-based approaches have been reported in Delphi-AHP studies in healthcare and public health, in which differences between mean expert ratings were converted into Saaty values using comparable interval-based rules [37–39]. In the present study, the 0.25-point intervals were therefore used as a study-specific mapping rule for constructing the judgment matrices. However, these specific cutoffs are not prescribed by conventional AHP methodology, and the cited studies represent applications of similar conversion procedures rather than methodological validation of these exact thresholds. The robustness of the resulting priorities was therefore further examined through sensitivity analysis.

Based on the converted pairwise values, judgment matrices were constructed for indicators within each hierarchical level. Local priorities were estimated using the principal eigenvector method, and global weights were obtained by multiplying each local weight by the corresponding parent-domain weight. The maximum eigenvalue, consistency index (CI), and consistency ratio (CR) were calculated for each judgment matrix, with CR ≤ 0.10 considered acceptable according to conventional AHP criteria [40, 41]. Because converting continuous differences in mean importance scores into discrete Saaty categories may introduce threshold effects, sensitivity analyses were conducted to examine whether small changes in the conversion boundaries materially altered the resulting weights or indicator rankings.

**Table 1:** Criteria for converting delphi importance score differences into saaty scale values

Value	Saaty scale value	Interpretation
I_m_ - I_n_ = 0.00	1	m and n with equal importance
0.00 < │I_m_ - I_n_│ ≤ 0.25	2	m is between equally and slightly more important than n
0.25 < │I_m_ - I_n_│ ≤ 0.50	3	m is slightly more important than n
0.50 < │I_m_ - I_n_│ ≤ 0.75	4	m is between slightly and moderately more important than n
0.75 < │I_m_ - I_n_│ ≤ 1.00	5	m is more important than n
1.00 < │I_m_ - I_n_│ ≤ 1.25	6	m is between moderately and strongly more important than n
1.25 < │I_m_ - I_n_│ ≤ 1.50	7	m is obviously more important than n
1.50 < │I_m_ - I_n_│ ≤ 1.75	8	m is between strongly and extremely more important than n
│I_m_ - I_n_│ > 1.75	9	m is absolutely more important than n

Note: I_m_ and I_n_ denote the second-round mean importance scores of indicators m and n, respectively. The Saaty value was determined according to │I_m_ - I_n_│. When I_m_ > I_m_, the corresponding scale value was used directly; when I_m_ < I_n_, its reciprocal was used. The 0.25-point intervals were predefined for converting differences in the 5-point importance scores into pairwise judgment values

### Data analysis

AHP weight calculation was conducted in Microsoft Excel using judgment matrices derived from the second-round Delphi importance scores. Local priorities were estimated using the principal eigenvector method, and global weights were obtained by multiplying each local weight by the corresponding parent-level weight. Matrix consistency was assessed using the consistency ratio, with CR ≤ 0.10 indicating acceptable consistency [40, 41].

To assess the robustness of the score-to-Saaty conversion, sensitivity analyses were performed by introducing small perturbations to the predefined conversion boundaries, drawing on established approaches in AHP research for evaluating the stability of priority estimates under variations in pairwise judgments [42, 43]. The internal cutoff points were shifted by ± 0.05 points while preserving their ordinal sequence and the overall 1–9 scale structure. For each alternative specification, the judgment matrices, local weights, and global weights were recalculated. Robustness was evaluated by comparing the baseline and perturbed scenarios in terms of absolute changes in indicator weights, preservation of the highest-ranked indicators, and Spearman rank correlations between the resulting indicator rankings.

## Results

### Expert panel characteristics

A total of 52 experts were invited to participate in the first round of Delphi consultation, and all 52 returned valid questionnaires, yielding a response rate of 100.00%. The same 52 experts were invited to participate in the second round, of whom 48 returned valid questionnaires, yielding a response rate of 92.31%. The expert panel included professionals from clinical nursing services, nursing management, internet hospital operation, nursing education, and health information management. Most participants had more than 10 years of professional experience, held a bachelor’s degree or higher, and had experience related to Internet Plus Nursing Services. The professional and demographic characteristics of the second-round participants were broadly comparable with those of the first-round panel, indicating that the multidisciplinary composition of the expert panel was largely maintained across the two consultation rounds. Detailed characteristics of participants in both rounds are presented in Table 2.

**Table 2:** Characteristics of Delphi expert panel participants in the two rounds

Characteristic	Round 1 (*n* = 52), n (%)	Round 2 (*n* = 48), n (%)
Professional background		
Clinical nursing services	24(46.15)	22(45.83)
Nursing management	6(11.54)	6(12.50)
Internet hospital operation	4(7.69)	4(8.33)
Nursing education	12(23.08)	11(22.92)
Health information management	6(11.54)	5(10.42)
Years of professional experience		
5–10	6(11.54)	6(12.50)
11–20	23(44.23)	21(43.75)
21–30	21(40.38)	19(39.58)
≥ 31	2(3.85)	2(4.17)
Professional title		
Middle	32(61.54)	29(60.42)
Associate senior	17(32.69)	16(33.33)
Senior	3(5.77)	3(6.25)
Education level		
Bachelor’ degree	29(55.77)	27(56.25)
Master’ degree	10(19.23)	9(18.75)
Doctor’ degree	13(25.00)	12(25.00)
Experience with Internet Plus Nursing Services		
Yes	41(78.85)	38(79.17)
No	11(21.15)	10(20.83)

### Expert authority and consensus formation

The expert authority coefficient was 0.74 in the first round, with a judgment coefficient of 0.77 and a familiarity coefficient of 0.71, indicating an acceptable level of expert authority. Kendall’s W was used to assess the degree of agreement among experts during the Delphi process.The five first-level domains were specified a priori and were not subject to structural screening or modification during the Delphi rounds. Experts nevertheless rated their importance. Because only five first-level indicators were included, Kendall’s W at this level was interpreted descriptively rather than inferentially, as the conventional chi-square approximation is less reliable when the number of ranked items is small. Consensus assessment therefore focused primarily on the second- and third-level indicators.

Kendall’s W for the second-level indicators increased from 0.150 in the first round to 0.196 in the second round, while Kendall’s W for the third-level indicators increased from 0.144 to 0.205. All tests for the second- and third-level indicators were statistically significant (*P* < 0.001). Although the absolute W values remained relatively low, indicating limited concordance in the relative ranking of indicators, the higher W values observed in the second round suggest that expert judgments became more convergent following structured feedback and indicator refinement. Therefore, the Delphi process demonstrated improved, although still limited, agreement across rounds (Table 3).

**Table 3:** Kendall’s coefficients of concordance for Delphi expert ratings

Hierarchical level	Kendall W	Chi-square(df)	*P* value
First round			
First level	0.034	Not applicable	Not applicable
Second level	0.150	116.704(15)	<0.001
Third level	0.144	359.026(48)	<0.001
Second round			
First level	0.120	Not applicable	Not applicable
Second level	0.196	122.323(13)	<0.001
Third level	0.205	413.993(42)	<0.001

Note: Kendall’s W for the five first-level indicators was reported descriptively because the small number of ranked items limits the reliability of the conventional chi-square approximation; consensus was therefore interpreted primarily for the second- and third-level indicators. Although the absolute W values for the second- and third-level indicators remained relatively low, their increases from the first to the second round indicated improved convergence of expert judgments

### Refinement of readiness indicators

Based on the predefined inclusion criteria and expert feedback, the preliminary indicator framework was revised after the first Delphi round. At the second level, 4 indicators were modified and 2 were deleted. At the third level, 8 indicators were deleted, 10 were modified, 4 were merged into 2, and 2 new indicators were added. These revisions clarified the scope of public hospital-led digital home nursing services and strengthened indicators related to nurse practice protection, digital platform support, payment arrangements, patient safety, service accessibility, and accountability. In the second Delphi round, all retained indicators met the predefined inclusion criteria. The final framework comprised 5 first-level domains, 14 s-level indicators, and 43 third-level indicators, covering Service Access and Institutional Requirements, Workforce and Digital Platform Infrastructure, Patient Adoption, Payment, and Value Recognition, Substitute-Care Boundaries and Service Continuity, and Operational Quality, Safety, and Accountability **(**Table 4**)**.

**Table 4:** Final domains, indicators, and global weights for public hospital-led digital home nursing

First-level indicators	Second-level indicators	Weights	Third-level indicators	Weights
1 Service Access and Institutional Requirements (0.1405)	1.1 Level of policy access and institutional constraints for “Internet Plus Nursing Service”	0.0937	1.1.1 Threshold of policy conditions for market entry	0.0131
			1.1.2 Degree of regulatory stringency regarding nurse practice qualifications	0.0312
			1.1.3 Extent of restrictions on multi-site nurse practice policies	0.0495
	1.2 Level of platform construction and operational costs for “Internet Plus Nursing Service”	0.0468	1.2.1 Level of information platform construction and maintenance costs	0.0074
			1.2.2 Level of equipment and facility investment costs	0.0278
			1.2.3 Level of marketing and patient acquisition costs	0.0117
2. Workforce and Digital Platform Infrastructure (0.3229)	2.1 Level of nursing human resource supply and stability in “Internet Plus Nursing Service”	0.2152	2.1.1 Nurses’ willingness to participate in the service	0.0583
			2.1.2 Rationality of nurse income distribution mechanism	0.0259
			2.1.3 Degree of specialized nursing manpower supply guarantee	0.0900
			2.1.4 Adequacy of risk protection mechanisms for nurses’ practice	0.0410
	2.2 Degree of technological platform support and resource dependence in “Internet Plus Nursing Service”	0.1076	2.2.1 Capability of the technological platform to support nursing services	0.0320
			2.2.2 Level of platform system stability and data security assurance	0.0581
			2.2.3 Degree of dependence on external technological resources	0.0176
3. Patient Adoption, Payment, and Value Recognition (0.2447)	3.1 Patients’ price sensitivity toward “Internet Plus Nursing Service”	0.0342	3.1.1 Proportion of out-of-pocket expenses borne by patients	0.0228
			3.1.2 Price level relative to offline nursing services	0.0114
	3.2 Patients’ perceived value of “Internet Plus Nursing Service”	0.0814	3.2.1 Patients’ recognition of the convenience and accessibility value of the service	0.0271
			3.2.2 Patients’ recognition of the standardization, safety, and trustworthiness of the service	0.0114
			3.2.3 Patients’ recognition of the comprehensive advantages of this service compared to other care modalities	0.0429
	3.3 Level of medical insurance payment support for “Internet Plus Nursing Service”	0.1292	3.3.1 Coverage of “Internet Plus Nursing Service” items under the medical insurance reimbursement scope	0.0861
			3.3.2 Level of medical insurance reimbursement rate support	0.0431
4. Substitute-Care Boundaries and Service Continuity (0.1065)	4.1 Accessibility of traditional nursing services	0.0632	4.1.1 Availability of offline nursing services at physical medical institutions	0.0197
			4.1.2 Prevalence of home hospital beds and related home-based medical care services	0.0124
			4.1.3 Substitutability of basic care needs by caregivers or other non-professional care providers	0.0312
	4.2 Substitution capability of non-public platforms	0.0266	4.2.1 Level of diversification of service items offered by non-public platforms	0.0166
			4.2.2 Capability of non-public platforms to provide continuous nursing services	0.0036
			4.2.3 Patients’ recognition of the service quality of non-public platforms	0.0063
	4.3 Substitution capability of community and elderly care institutions	0.0167	4.3.1 Feasibility of substituting with community nursing services	0.0027
			4.3.2 Feasibility of substituting with nursing services in elderly care institutions	0.0050
			4.3.3 Feasibility of substituting with new care models	0.0090
5. Operational Quality, Safety, and Accountability (0.1854)	5.1 Level of service differentiation in “Internet Plus Nursing Service”	0.0770	5.1.1 Degree of differentiation in specialized nursing service items	0.0151
			5.1.2 Degree of differentiation in the scope of service coverage	0.0239
			5.1.3 Level of personalized nursing service provision	0.0380
	5.2 Level of cost control and pricing competitiveness of nursing services	0.0198	5.2.1 Extent of nursing labor cost constraints	0.0047
			5.2.2 Extent of platform operation cost constraints	0.0124
			5.2.3 Rationality of nursing service pricing	0.0027
	5.3 Level of service quality and operational efficiency	0.0343	5.3.1 Timeliness of service response	0.0094
			5.3.2 Efficiency of nursing service delivery	0.0023
			5.3.3 Efficiency of service issue resolution	0.0125
			5.3.4 Level of personal safety protection for nursing staff	0.0067
			5.3.5 Level of patient safety protection during nursing care	0.0034
	5.4 Level of service accessibility and coverage	0.0543	5.4.1 Public awareness of the service through promotion and publicity	0.0161
			5.4.2 Convenience of mobile-based appointment booking	0.0089
			5.4.3 Accessibility of the service for special populations (elderly, disabled, and those with limited mobility)	0.0293

Note: The five first-level readiness domains were derived from the contextualized Porter’s Five Forces framework: Service Access and Institutional Requirements from the threat of new entrants; Workforce and Digital Platform Infrastructure from supplier power; Patient Adoption, Payment, and Value Recognition from buyer power; Substitute-Care Boundaries and Service Continuity from the threat of substitutes; and Operational Quality, Safety, and Accountability from industry rivalry. All weights presented are global weights and are reported to four decimal places. Minor discrepancies in summed values may occur because individual weights were rounded independently

**Fig. 3:**
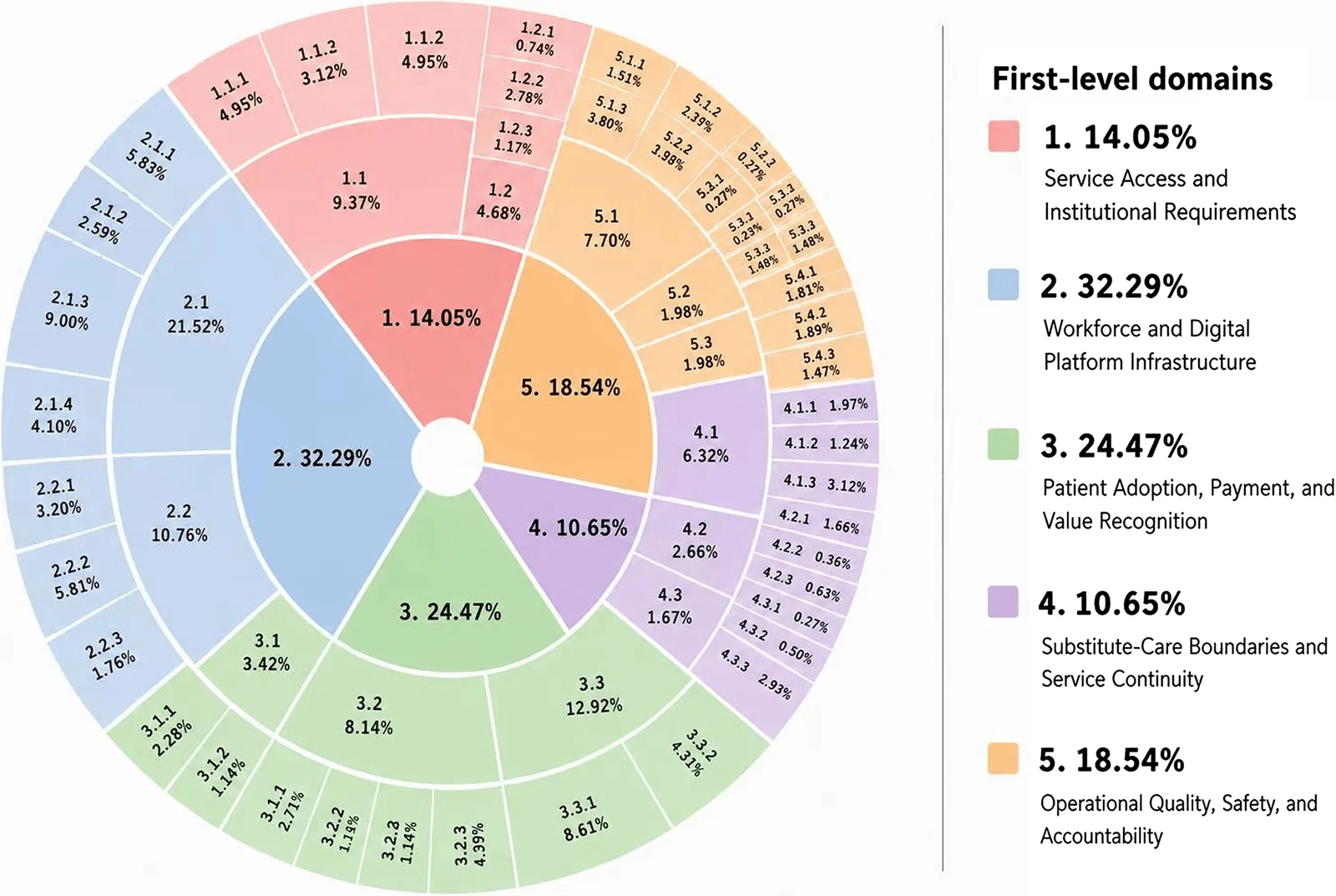
Hierarchical distribution of priority domains for public hospital-led internet plus nursing services

### Priority weights of the final readiness framework

**Fig. 4:**
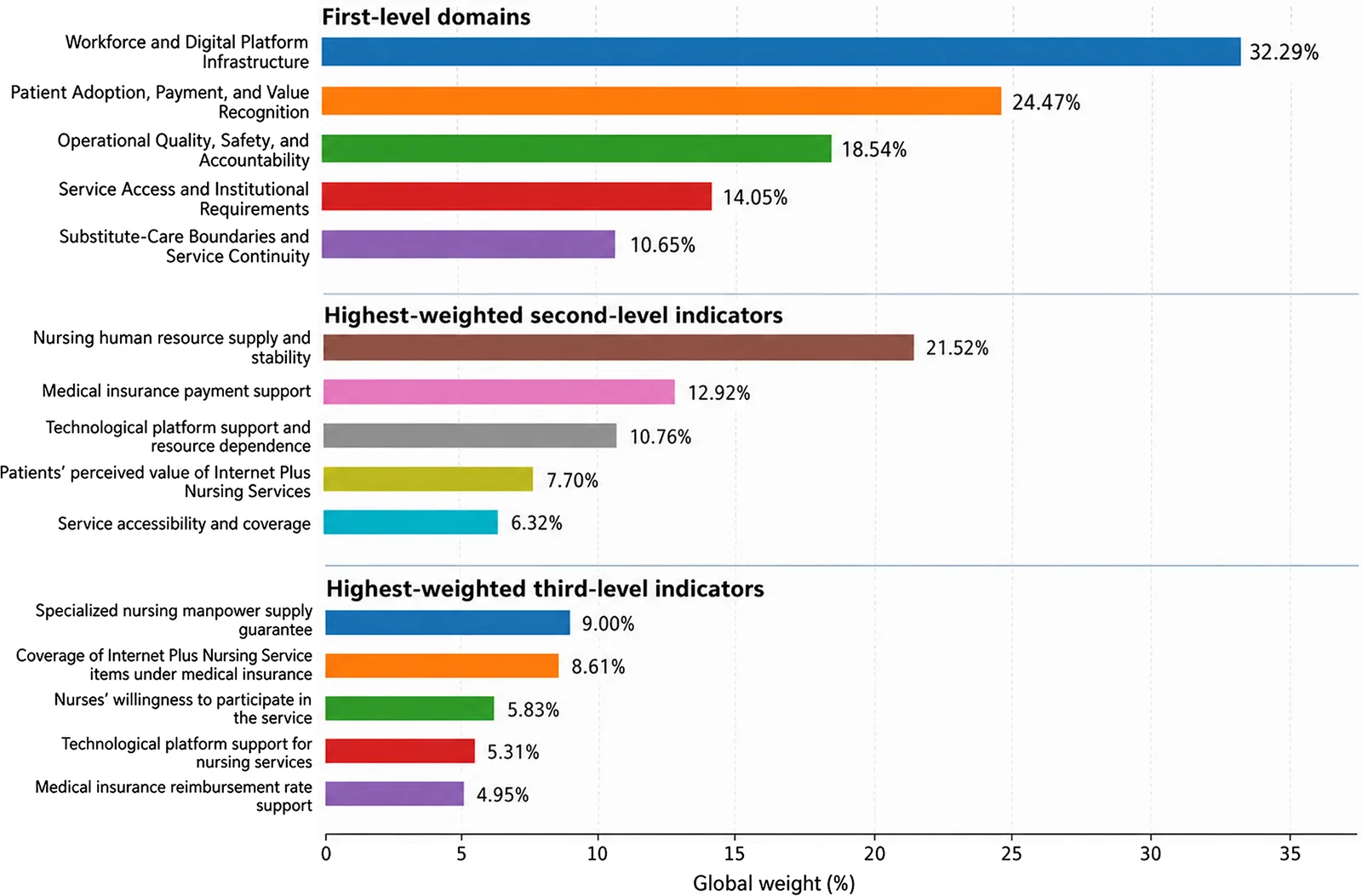
Priority weight profile of the digital home nursing framework

The final global weights were calculated using the Delphi-derived AHP procedure based on the second-round importance ratings from the 48 experts who completed the consultation. The five first-level domains were derived from the contextualized Porter’s Five Forces framework and operationalized as system-level readiness domains for public hospital-led digital home nursing.All AHP judgment matrices met the predefined consistency criterion. Among matrices requiring consistency testing, CR values ranged from 0.0088 to 0.0516, all below the prespecified threshold of 0.10; CR was not applicable to two-element comparison matrices because these are inherently consistent. Detailed consistency statistics for each judgment matrix are provided in Supplementary 4 Table S11.The final domains, indicators, and corresponding global weights are presented in Table 4, while their hierarchical distribution is shown in Fig. 3 and the overall priority profile is summarized in Fig. 4.

Among the five first-level domains, Workforce and Digital Platform Infrastructure had the highest global weight (32.29%), followed by Patient Adoption, Payment, and Value Recognition (24.47%), Operational Quality, Safety, and Accountability (18.54%), Service Access and Institutional Requirements (14.05%), and Substitute-Care Boundaries and Service Continuity (10.65%). At the second level, nursing human resource supply and stability had the highest global weight (21.52%), followed by medical insurance payment support (12.92%) and technological platform support and resource dependence (10.76%). At the third level, the highest-weighted indicators were specialized nursing manpower supply guarantee (9.00%), coverage of Internet Plus Nursing Service items under the medical insurance reimbursement scope (8.61%), and nurses’ willingness to participate in the service (5.83%). These findings indicate that workforce capacity, payment support, and digital platform infrastructure were assigned the highest relative priorities within the final readiness framework.

### Sensitivity analysis of the score-to-saaty conversion

Sensitivity analyses were conducted by shifting the predefined score-to-Saaty conversion boundaries by − 0.05 and + 0.05 points and recalculating the corresponding judgment matrices and indicator weights. As shown in Table 5, the ranking of the five first-level domains remained unchanged under both perturbation scenarios. Workforce and Digital Platform Infrastructure remained the highest-priority domain, followed by Patient Adoption, Payment, and Value Recognition and Operational Quality, Safety, and Accountability. Although the absolute weights showed some variation, the maximum change was 0.0358 and did not alter the ordering of the five domains.

The stability of the rankings across hierarchical levels is summarized in Table 6. Spearman rank correlations between the baseline and perturbed rankings were 1.000 at the first level, 0.969 and 1.000 at the second level, and 0.984 and 0.996 at the third level for the − 0.05 and + 0.05 scenarios, respectively. The maximum absolute changes in global weight were 0.0358, 0.0238, and 0.0179 across the three levels. All 10 highest-ranked third-level indicators in the baseline analysis remained within the top 10 under both perturbation scenarios, although several closely ranked indicators exchanged positions. All recalculated judgment matrices remained within the predefined consistency criterion, with the maximum CR below 0.10. Detailed indicator-level results are provided in Supplementary 4 Table S12.

**Table 5:** Sensitivity analysis of first-level domain weights and rankings under alternative conversion boundaries

First-level domain	Baseline weight	Baseline link	−0.05 weight	−0.05 rank	+ 0.05 weight	+ 0.05 rank	Maximum absolute weight change
Service Access and Institutional Requirements	0.1405	4	0.1130	4	0.1405	4	0.0276
Workforce and Digital Platform Infrastructure	0.3229	1	0.3586	1	0.3229	1	0.0358
Patient Adoption, Payment, and Value Recognition	0.2447	2	0.2709	2	0.2447	2	0.0262
Substitute-Care Boundaries and Service Continuity	0.1065	5	0.0853	5	0.1065	5	0.0212
Operational Quality, Safety, and Accountability	0.1854	3	0.1722	3	0.1854	3	0.0132

Note: In the sensitivity analyses, all internal score-to-Saaty conversion boundaries were shifted simultaneously by − 0.05 or + 0.05 points relative to the baseline specification. Maximum absolute change represents the largest absolute difference between the baseline weight and either perturbed scenario

**Table 6:** Stability of indicator rankings across hierarchical levels under alternative conversion boundaries

Hierachical level	No. of indicators	Spearman ρ (-0.05)	Spearman ρ (+ 0.05)	Maximum absolute Weight change
First level	5	1.000	1.000	0.0358
Second level	14	0.969	1.000	0.0238
Third level	43	0.984	0.996	0.0179

Note: Maximum absolute weight change represents the largest absolute difference from the baseline global weight across the two perturbation scenarios

## Discussion

### Principal findings

This study developed a readiness evaluation framework for public hospital-led Internet Plus Nursing Services by integrating a contextualized Porter’s Five Forces model, Delphi expert consultation, and analytic hierarchy process weighting. The five first-level domains were predefined through the contextualization of Porter’s Five Forces, whereas the second- and third-level indicators were refined through two rounds of Delphi consultation. The final framework comprised 5 first-level domains, 14 s-level indicators, and 43 third-level indicators. The expert authority coefficient was 0.74. Kendall’s W values for both second- and third-level indicators increased from the first to the second Delphi round, and all tests were statistically significant. However, the absolute W values remained relatively low, indicating limited concordance in the exact relative ranking of indicators. Importantly, the consistent increases in W across hierarchical levels suggest that structured feedback and iterative indicator refinement contributed to greater convergence of expert judgments in the second round. Therefore, the Delphi findings should be interpreted as showing improved convergence rather than a high absolute level of agreement.

All derived AHP judgment matrices met the predefined consistency criterion, indicating acceptable internal consistency of the resulting pairwise matrices. Nevertheless, the weighting results should be interpreted in light of the score-informed construction of these matrices. Unlike conventional AHP, the present study derived pairwise values from differences in the second-round Delphi importance scores rather than from direct pairwise comparisons. Accordingly, the consistency ratios reflect the internal coherence of the derived matrices rather than the validity of the score-to-Saaty conversion itself. Sensitivity analyses further showed that shifting the conversion boundaries by − 0.05 and + 0.05 points did not materially alter the overall priority structure. The five first-level domain rankings remained unchanged, while the second- and third-level rankings remained highly consistent with the baseline results. Although several closely ranked lower-level indicators exchanged positions, the main high-priority indicators were preserved across the alternative specifications.

The relatively low absolute Kendall’s W values warrant caution when interpreting the precise ranking of individual indicators. The expert panel represented multiple professional backgrounds, including clinical nursing, nursing management, internet hospital operation, nursing education, and health information management, and these different professional perspectives may have contributed to variation in the relative importance assigned to individual readiness dimensions. Thus, the final indicator weights should not be interpreted as reflecting strong unanimity regarding the exact ordering of indicators. Rather, they represent relative priority estimates derived from a multidisciplinary expert panel whose judgments became more convergent after the second Delphi round. Further empirical validation is needed to assess the stability of these rankings across different settings and data sources.

The weighting results showed that Workforce and Digital Platform Infrastructure received the highest first-level weight (32.29%), followed by Patient Adoption, Payment, and Value Recognition (24.47%), Operational Quality, Safety, and Accountability (18.54%), Service Access and Institutional Requirements (14.05%), and Substitute-Care Boundaries and Service Continuity (10.65%). This distribution suggests that readiness for public hospital-led digital home nursing is shaped primarily by the capacity to maintain an adequate nursing workforce and reliable digital platform infrastructure, together with patient adoption, payment support, and mechanisms for operational quality, safety, and accountability. The relatively lower weights assigned to Service Access and Institutional Requirements and Substitute-Care Boundaries and Service Continuity should not be interpreted as indicating that these domains are unimportant, but rather that they were assigned lower relative priority within the current expert-derived weighting structure.

At the second level, nursing human resource supply and stability had the highest global weight (21.52%), followed by medical insurance payment support (12.92%) and technological platform support and resource dependence (10.76%). At the third level, the highest-weighted indicators were specialized nursing manpower supply guarantee (9.00%), coverage of Internet Plus Nursing Service items under the medical insurance reimbursement scope (8.61%), and nurses’ willingness to participate in the service (5.83%). These findings place workforce capacity, reimbursement coverage, and nurse participation among the most prominent readiness conditions identified by the expert panel. They further suggest that readiness for digital home nursing depends not only on the availability of digital service channels, but also on whether hospitals can sustain sufficient professional nursing resources, provide reliable platform support, and establish payment arrangements that facilitate continued service use.

Taken together, the findings support viewing Internet Plus Nursing Services as a platform-mediated and organizationally distributed model of nursing care rather than simply an extension of hospital-based nursing into the home. The framework highlights the interdependence of workforce capacity, digital infrastructure, payment support, patient adoption, service boundaries, and quality and accountability. However, because the first-level structure was theoretically predefined and the observed Delphi concordance remained modest in absolute terms, the resulting priorities should be interpreted cautiously and considered subject to further empirical validation. The following sections therefore discuss how these domains relate to system readiness, workforce and platform support, payment and access, quality and accountability, and the organization of professional nursing care outside hospital settings.

### From service expansion to system readiness

Building on the priority pattern identified above, the evaluation of Internet Plus Nursing Services can be reframed from service expansion toward system readiness. In the early development of digital home nursing, service availability, platform functions, patient satisfaction, and nurses’ willingness to participate are necessary evaluation concerns. However, these dimensions do not fully capture whether a public hospital-led digital home nursing system is prepared to deliver care safely, continuously, and accountably across institutional and home settings. The concentration of higher weights in workforce protection, digital platform infrastructure, payment support, quality monitoring, and accountability mechanisms indicates that Internet Plus Nursing Services should be assessed as a platform-mediated care system rather than as a collection of individual service items.

The resulting priority structure also aligns with the health-system and implementation perspectives considered during framework selection. Digital health readiness research emphasizes that implementation depends not only on technological availability but also on the readiness of users, organizations, workflows, and wider service systems [44, 45]. In technology-enabled care, readiness is therefore not a single technical attribute; it reflects whether the clinical workforce, digital infrastructure, financing arrangements, patients, organizations, and regulatory environment are sufficiently aligned to support implementation and sustainability. Sociotechnical implementation perspectives further suggest that the adoption and scale-up of digital health technologies are shaped by interactions among the technology, the value proposition, adopters, organizations, and the broader system context [28]. These perspectives are particularly relevant to digital home nursing, where care is delivered outside the hospital but remains dependent on hospital-based professional standards, platform-supported coordination, payment rules, patient trust, and accountability mechanisms.

From this perspective, the contextualized Five Forces framework developed in this study should be understood as a structural approach for organizing system-readiness pressures in a multi-actor digital care environment. Its contribution is not to reproduce a market-competition analysis, but to translate these pressures into five assessable readiness domains: Service Access and Institutional Requirements; Workforce and Digital Platform Infrastructure; Patient Adoption, Payment, and Value Recognition; Substitute-Care Boundaries and Service Continuity; and Operational Quality, Safety, and Accountability.

### Workforce, platform infrastructure, and payment as core enablers

Within this system-readiness framework, Workforce and Digital Platform Infrastructure and Patient Adoption, Payment, and Value Recognition emerged as two closely connected enabling domains rather than separate implementation concerns. Together, they form a practical readiness chain for public hospital-led digital home nursing: the nursing workforce determines whether professional care can be safely supplied; the digital platform determines whether dispersed care can be coordinated, documented, monitored, and traced; and payment arrangements determine whether patients can afford, access, and recognize the value of professional home-based nursing. This interpretation is consistent with recent work on virtual nursing, which emphasizes that technology-enabled nursing models depend on workforce sustainability, role redesign, workflow integration, and organizational support rather than technology availability alone [46].

The Workforce and Digital Platform Infrastructure domain is particularly important in digital home nursing because care is delivered outside the controlled institutional environment while remaining dependent on hospital-based professional standards. Nurses providing Internet Plus Nursing Services face dispersed work settings, limited direct supervision, and potential occupational, legal, and communication risks. Thus, workforce readiness involves not only sufficient staffing, but also qualification management, specialized training, workload allocation, occupational safety, liability protection, and emergency response support. At the same time, the platform is not simply an appointment or dispatch tool. It functions as part of the governance infrastructure that makes home-based nursing visible and auditable through identity verification, service matching, informed consent, standardized documentation, data security, quality monitoring, complaint handling, and accountability tracing. This platform-governance interpretation is supported by nurse-led telehealth research, which shows that digitally mediated nursing services require integration of professional roles, access pathways, clinical workflows, and organizational support to improve service reach and continuity [47].

Within the Patient Adoption, Payment, and Value Recognition domain, payment support extends this readiness chain from the supply and coordination side to patient access and sustained service use. The high priority assigned to medical insurance payment support indicates that affordability is not only a demand-side issue but also a condition for equitable and sustained implementation. A recent scoping review of telehealth reimbursement found that health insurance payment has become an important policy mechanism for expanding telehealth access and integrating digitally mediated care into routine health systems [48]. At the same time, digital health equity research cautions that digital services may reproduce or widen inequalities when financing, access, and implementation arrangements are insufficiently inclusive [49]. For Internet Plus Nursing Services, insufficient payment support may turn professional home-based nursing into a selective convenience service rather than an accessible component of continuing care for older adults, patients with chronic diseases, postdischarge patients, and people with limited mobility. In this sense, workforce protection, platform infrastructure, and payment support jointly determine whether digital home nursing can move from service availability to sustainable use. In this sense, Workforce and Digital Platform Infrastructure and Patient Adoption, Payment, and Value Recognition represent interconnected readiness domains that jointly influence whether digital home nursing can progress from service availability to sustainable use.

### Quality, accountability, and care boundaries in distributed home nursing

However, even when workforce, platform, and payment conditions are in place, digital home nursing still requires mechanisms to ensure that dispersed care remains clinically safe, professionally bounded, and institutionally accountable. This is because Internet Plus Nursing Services shift professional nursing from the hospital to the home, where direct institutional supervision is weaker, care environments are less standardized, and service processes may involve patients, family caregivers, platform operators, hospitals, and community providers. Recent evidence on telehealth in home care shows that specialized nurses play a central role in maintaining patient safety, but that safety depends on clearly defined roles, structured communication, risk identification, patient education, and escalation pathways [50]. Therefore, quality governance in digital home nursing should not be limited to post hoc patient satisfaction or complaint handling; it should be embedded throughout the service process.

In this context, platform-enabled documentation and traceability become central to accountability. Digital home nursing services require more than an online appointment system: they require verifiable provider identity, service authorization, informed consent, standardized nursing records, time-and-location documentation, risk reporting, data security, and mechanisms for reviewing adverse events or disputes. A recent scoping review of digital home care interventions for older adults found that digital interventions have the potential to improve access, communication, continuity, and quality of home-based primary care, but also noted challenges related to inequalities, information exchange, and integration with care processes [51]. This supports the interpretation that digital platforms should be evaluated not only by usability or convenience, but by whether they can make dispersed care visible, auditable, and connected to institutional quality systems.

Care boundaries are another essential part of quality governance. Home-based care is often delivered within a mixed ecosystem that may include hospitals, primary care institutions, community services, elderly care organizations, commercial platforms, care aides, and family caregivers. These actors may complement professional nursing services, but they may also blur responsibility when the scope of practice, referral criteria, and escalation pathways are unclear. A systematic review of hospital-at-home care similarly emphasized that home-based models require clearly defined components and processes to support implementation and planning [52]. For Internet Plus Nursing Services, the substitute-care issue should therefore not be interpreted only as competition from other providers, but as a boundary-governance problem: health systems must clarify which tasks require registered nurses, which tasks can be delegated or supplemented, and how professional responsibility is maintained when care is delivered beyond hospital walls. This boundary logic also links the China-based framework to broader debates on how digital home care systems can remain safe, governable, and transferable across different health care context.

### International relevance and transferability of the framework

The need to govern digital home nursing as a structured health information system is also reflected in international home-based care models. Although these models differ in clinical scope and payment arrangements, they reveal similar system pressures to those identified in this study. In the United States, the Acute Hospital Care at Home initiative allows certain Medicare-certified hospitals to provide inpatient-level care in patients’ homes, highlighting the importance of institutional eligibility, hospital responsibility, and payment authorization when care is moved outside the hospital [53]. In England, virtual wards, also known as hospital-at-home services, allow patients to receive hospital-level care at home and are used to support care in patients’ usual place of residence, including care homes, which underscores the importance of discharge linkage, remote monitoring, community coordination, and escalation pathways [54]. In Victoria, Australia, the Victorian Virtual Emergency Department has demonstrated the importance of clinical governance, digitally enabled workforce, integrated service delivery, and quality assurance in supporting safe virtual care delivery [55]. These examples suggest that digital home-based care is not merely a technological extension of hospital services, but a system redesign process involving provider qualification, workforce capacity, platform-supported coordination, payment rules, quality governance, and accountability.

For China’s Internet Plus Nursing Services, the relevance of these international cases lies less in direct model replication than in their shared governance logic. Compared with hospital-at-home models in the United States and England, China’s digital home nursing services are more nursing-procedure oriented and more strongly organized through public hospitals and internet hospital platforms. This creates a distinctive opportunity to integrate professional nursing services, platform documentation, and institutional accountability within a public hospital-led system. At the same time, international experience suggests several areas in which China’s model could be strengthened: clearer institutional responsibility and reimbursable service categories, closer linkage with discharge planning and primary care follow-up, more standardized escalation pathways, and stronger platform-based monitoring of quality, safety, adverse events, data security, and complaints. The broader implication is that China’s digital home nursing services should not be evaluated only by platform availability or service volume. They should be developed as a governable digital care system in which workforce protection, payment support, service-boundary management, clinical governance, and accountability tracing are built into the service architecture.

### Limitations

This study has several limitations. First, the five first-level readiness domains were defined a priori by the research team through contextualization of Porter’s Five Forces model and were not subjected to the same structural screening and refinement process as the second- and third-level indicators. Although the lower-level indicators were subsequently screened and refined through two rounds of expert consultation, the top-level structure was primarily theory-driven and therefore partly reflects the research team’s conceptual judgment regarding how Porter’s Five Forces should be adapted to digital home nursing. Future studies should further examine the content and structural validity of these first-level domains and assess whether alternative domain structures may better represent readiness in different health care contexts.

Second, the Delphi expert panel was situated within the Chinese health care context, and the relative importance of indicators may differ across regions or countries with different nursing scopes of practice, reimbursement systems, digital infrastructure, home care regulations, and models of hospital-community coordination. Therefore, the framework should be further examined before being applied to other health system contexts.

Third, the priority weights were derived from expert judgment rather than real-world service performance data. Although Kendall’s W increased from the first to the second Delphi round, indicating greater convergence of expert judgments, the absolute values remained relatively low. Therefore, the resulting weights should be interpreted as relative priority estimates rather than definitive rankings supported by strong expert unanimity. Future studies should validate the framework and its weighting structure using multicenter samples, platform operation data, service utilization records, nurse safety events, patient-reported outcomes, and other empirical quality indicators.

Fourth, the AHP judgment matrices were constructed indirectly from differences in the second-round Delphi importance scores rather than from direct pairwise comparisons. The score-to-Saaty conversion provided an operational approach for translating the Delphi ratings into relative priority estimates, but discretizing continuous differences in mean scores may introduce threshold effects when indicator scores are closely spaced. Sensitivity analyses showed that modest shifts in the predefined conversion boundaries did not materially alter the overall priority structure, although minor rank changes occurred among several closely weighted lower-level indicators. The resulting weights should therefore be interpreted with appropriate caution. Future studies could compare this weighting approach with direct pairwise AHP elicitation or alternative weighting methods to further examine the stability of the priority structure.

Finally, AHP assumes relative independence among indicators, whereas workforce stability, platform capacity, payment support, patient adoption, quality monitoring, and accountability mechanisms may interact in actual implementation. Future research could use empirical modeling, network-based weighting approaches, or mixed-methods evaluation to examine the interdependencies among these domains and to further assess how the framework performs in real-world implementation settings.

## Conclusions

This study developed a readiness evaluation framework for public hospital-led digital home nursing services in China, using Internet Plus Nursing Services as the empirical context. By integrating literature review, policy document analysis, Delphi expert consultation, and score-informed AHP weighting, the study established a hierarchical framework comprising 5 first-level domains, 14 s-level indicators, and 43 third-level indicators. The framework extends the assessment of digital home nursing beyond service availability and platform functionality by organizing readiness around service access and institutional requirements, workforce and digital platform infrastructure, patient adoption and payment, substitute-care boundaries and service continuity, and operational quality, safety, and accountability.

The weighting results identified Workforce and Digital Platform Infrastructure as the highest-priority domain, followed by Patient Adoption, Payment, and Value Recognition and Operational Quality, Safety, and Accountability. These findings indicate that readiness for digital home nursing depends not only on the availability of digital service channels, but also on whether professional nursing resources, platform support, payment arrangements, service boundaries, quality assurance, and accountability mechanisms can be coordinated within a safe and sustainable service system. The stability of the overall priority structure under the sensitivity analyses further supports the robustness of the resulting prioritization, although the weights should be interpreted as relative priority estimates derived from expert judgment rather than definitive performance-based rankings.

The proposed framework provides a structured reference for public hospitals, platform operators, and health service regulators seeking to assess and strengthen readiness for digital home nursing. Further research should validate the framework across multiple institutions and health system contexts using real-world operational, service utilization, nurse safety, patient-reported, and other empirical performance data.

## Supplementary Information

Below is the link to the electronic supplementary material.

Supplementary Material 1: Supplementary Material 1 provides the literature search modules, database-specific search strategies, eligibility criteria, and procedures for study screening and indicator extraction (Table S1).

Supplementary Material 2: Supplementary Material 2 presents the distribution and complete list of the national and local policy documents included in the policy analysis (Tables S2–S9).

Supplementary Material 3: Supplementary Material 3 provides the quantification criteria, standardization procedure, and calculation method for the expert authority coefficient (Table S10).

Supplementary Material 4: Supplementary Material 4 presents the AHP consistency-testing results, including the maximum eigenvalue, consistency index, and consistency ratio for each judgment matrix (Table S11), together with detailed sensitivity-analysis results for the second- and third-level indicator weights and rankings under alternative score-to-Saaty conversion boundaries (Table S12).

Supplementary Material 5: Supplementary Material 5 includes the first-round Delphi expert consultation questionnaire.

Supplementary Material 6: Supplementary Material 6 includes the second-round Delphi expert consultation questionnaire.

## Data Availability

The data generated and analyzed during this study are available from the corresponding author upon reasonable request. The search strategy, included policy documents, expert authority scoring criteria, and AHP consistency test results are provided in the supplementary materials.
