# Supplementary material for: Developing a readiness evaluation framework for public hospital-led digital home nursing services in China: a Delphi-AHP study
Source: BMC Nurs. 2026 Aug 29;25:865. doi: 10.1186/s12912-026-05311-6 (PMC13589386; doi:10.1186/s12912-026-05311-6)
Supplement: Supplementary file 1 [file 12912_2026_5311_MOESM1_ESM.docx]

## Supplementary material 1

## Search Strategy

## Database sources

## A structured literature search was conducted in PubMed, China National Knowledge Infrastructure (CNKI), and Wanfang Database to identify studies relevant to indicator extraction. The search covered studies published from database inception to January 1, 2026, and was limited to studies published in English or Chinese.

## Because this study contextualized Porter’s Five Forces model as an organizing framework for health information system readiness in digital home nursing, the search strategy was designed to capture evidence related to digital nursing services, home-based care, platform-mediated service delivery, service governance, patient safety, and evaluation indicator development. Terms related to service-system structure, provider-level performance, platform governance, digital health readiness, information quality, data security, system stability, quality monitoring, accountability, and service traceability were included to align the search strategy with the focus of the framework. The search strategy was organized into five topic modules. The search modules and main concepts are presented in **Table S1**.

## **Table S1.** Search Modules and Main Concepts Used for Literature Retrieval

| Search module | Main concepts |
| --- | --- |
| Digital nursing services | Internet Plus Nursing Services; internet-based nursing; digital nursing; mobile nursing; virtual nursing; telenursing |
| Home-based and continuing care | home-based nursing; home care nursing; door-to-door nursing; continuing nursing; community nursing; postdischarge nursing |
| Platform and health information system readiness | digital platform; platform governance; health information system readiness; digital health readiness; information quality; data security; system stability; service traceability |
| Governance and safety | service governance; nursing regulation; scope of practice; patient safety; quality monitoring; risk management; accountability |
| Evaluation framework and indicators | evaluation framework; indicator system; quality indicator; readiness assessment; service capacity; performance assessment; Delphi; analytic hierarchy process |

## **PubMed**

Search field: Title/Abstract

Search strategy:

(“internet plus nursing service”[Title/Abstract] OR “Internet Plus Nursing Service”[Title/Abstract] OR “internet-based nursing service”[Title/Abstract] OR “online nursing service”[Title/Abstract] OR “digital nursing”[Title/Abstract] OR “virtual nursing”[Title/Abstract] OR “mobile nursing”[Title/Abstract] OR “telenursing”[Title/Abstract] OR “tele-nursing”[Title/Abstract] OR “internet-based home care”[Title/Abstract] OR “home-based nursing”[Title/Abstract] OR “home care nursing”[Title/Abstract] OR “community nursing”[Title/Abstract] OR “postdischarge nursing”[Title/Abstract] OR “continuing nursing”[Title/Abstract])

AND

(“health information system readiness”[Title/Abstract] OR “digital health readiness”[Title/Abstract] OR “platform governance”[Title/Abstract] OR “digital platform”[Title/Abstract] OR “information quality”[Title/Abstract] OR “data security”[Title/Abstract] OR “system stability”[Title/Abstract] OR “service traceability”[Title/Abstract] OR “quality monitoring”[Title/Abstract] OR “healthcare governance”[Title/Abstract] OR “health care governance”[Title/Abstract] OR “service governance”[Title/Abstract] OR “nursing regulation”[Title/Abstract] OR “scope of practice”[Title/Abstract] OR “patient safety”[Title/Abstract] OR “risk management”[Title/Abstract] OR “accountability”[Title/Abstract] OR “provider performance”[Title/Abstract] OR “service-system”[Title/Abstract])

AND

(“evaluation framework”[Title/Abstract] OR “evaluation indicator”[Title/Abstract] OR “indicator system”[Title/Abstract] OR “quality indicator”[Title/Abstract] OR “readiness assessment”[Title/Abstract] OR “readiness framework”[Title/Abstract] OR “performance assessment”[Title/Abstract] OR “service capacity”[Title/Abstract] OR “Delphi”[Title/Abstract] OR “analytic hierarchy process”[Title/Abstract]

## **China National Knowledge Infrastructure**

Search field: Topic

Search strategy:

(“互联网+护理服务” OR “网约护理” OR “居家护理” OR “上门护理” OR “移动护理” OR “延续性护理” OR “社区护理”OR “远程护理”)

AND

(“信息系统准备度” OR “数字健康准备度” OR “平台治理” OR “数字平台” OR “信息质量” OR “数据安全” OR “系统稳定性” OR “服务可追溯” OR “质量监测” OR “服务治理” OR “护理监管” OR “执业范围” OR “患者安全” OR “风险管理” OR “责任追溯” OR “服务系统” OR “服务能力” OR “组织准备度”)

AND

(“评价指标” OR “指标体系” OR “评价框架” OR “服务能力评价” OR “服务质量评价” OR “绩效评价” OR “准备度评价” OR “准备度框架” OR “德尔菲法” OR “层次分析法”)

## **Wanfang database**

Search field: Topic or Keyword

Search strategy:

(“互联网+护理服务” OR “网约护理” OR “互联网护理” OR “数字护理” OR “移动护理” OR “远程护理” OR “居家护理” OR “上门护理” OR “延续性护理” OR “社区护理” OR “出院延续护理”)

AND

(“信息系统准备度” OR “数字健康准备度” OR “平台治理” OR “数字平台” OR “信息质量” OR “数据安全” OR “系统稳定性” OR “服务可追溯” OR “质量监测” OR “服务治理” OR “护理监管” OR “执业范围” OR “患者安全” OR “风险管理” OR “责任追溯” OR “服务系统” OR “服务能力” OR “组织准备度”)

AND

(“评价指标” OR “指标体系” OR “评价框架” OR “服务能力评价” OR “服务质量评价” OR “绩效评价” OR “准备度评价” OR “准备度框架” OR “德尔菲法” OR “层次分析法”)

## **Eligibility criteria**

## Studies were considered eligible if they met at least one of the following criteria:

## 1. Focused on Internet Plus Nursing Services, internet-based nursing, home-based nursing, mobile nursing, community nursing, telenursing, virtual nursing, digital nursing, continuing nursing, or postdischarge nursing services.

## 2. Discussed health information system readiness, digital health readiness, platform governance, service governance, nursing regulation, scope of practice, patient safety, quality monitoring, risk management, accountability, service capacity, or provider-level service performance in digital health, nursing services, home-based care, or medical institutions.

## 3. Developed or applied an evaluation framework, evaluation indicator system, quality indicator system, performance assessment framework, readiness assessment framework, or service evaluation framework relevant to nursing services, digital health services, home-based care, platform-mediated care, or hospital management.

## Studies were excluded if they were duplicates, news reports, conference abstracts without full text, unrelated clinical intervention studies, or studies that did not provide information relevant to indicator extraction.

## **Screening and indicator extraction**

Two researchers independently screened the retrieved records according to the inclusion and exclusion criteria. After duplicate records were removed, titles and abstracts were reviewed, followed by full-text assessment. Disagreements were resolved through discussion, and a third researcher was consulted when consensus could not be reached.

Potential indicators were extracted from included studies and classified according to the contextualized Porter’s Five Forces framework. During extraction, particular attention was paid to indicators related to service access, nursing workforce and platform resources, patient adoption and payment, substitute-care boundaries, platform functionality, data security, service traceability, quality monitoring, and accountability. Extracted indicators were then refined through policy document analysis and expert pre-consultation before being included in the initial indicator pool.
