# Supplementary material for: Developing a readiness evaluation framework for public hospital-led digital home nursing services in China: a Delphi-AHP study
Source: BMC Nurs. 2026 Aug 29;25:865. doi: 10.1186/s12912-026-05311-6 (PMC13589386; doi:10.1186/s12912-026-05311-6)
Supplement: Supplementary file 3 [file 12912_2026_5311_MOESM3_ESM.docx]

# Supplementary material 3

To evaluate the reliability of the expert consultation results, the expert authority coefficient (Cr) was used to quantify the credibility of expert judgments. The authority coefficient was determined by two components: the familiarity coefficient (Cs), which reflected experts’ self-assessed familiarity with the consultation topic, and the judgment coefficient (Ca), which reflected the extent to which expert judgments were based on theoretical analysis, practical experience, literature evidence, and intuitive judgment.

In this study, Cs was directly assigned according to experts’ self-rated familiarity with the consultation topic, with values ranging from 0.20 to 1.00. The raw Ca score was rated on a 3-point scale for each judgment basis. To ensure comparability between Ca and Cs, each raw Ca score was divided by 3 for standardization, and the mean of the four standardized scores was used as the final Ca value. The expert authority coefficient was calculated as Cr = (Ca + Cs) / 2. The detailed scoring criteria and calculation method are shown in **Table S10**.

**Table S10.** Quantification criteria and calculation method for the expert authority coefficient.

| Variable | Measurement domain | Rating and assigned score | Standardization | Calculation |
| --- | --- | --- | --- | --- |
| Cs | Expert familiarity with the consultation topic | Extremely familiar=1.00;  very familiar=0.80;  moderately familiar=0.60;  slightly familiar=0.40; unfamiliar=0.20 | Not applicable | Directly assigned according to expert self-assessment |
| Ca | Basis of expert judgment, including practical experience, theoretical analysis, literature evidence, and intuitive judgment | high influence=3;  moderate influence=2;  low influence=1 | Each raw score was divided by 3 | Mean of the four standardized scores |
| Cr | Expert authority coefficient | Not applicable | Not applicable | Cr = (Cs + Ca)/2 |

Note. Cs represents the familiarity coefficient, Ca represents the judgment coefficient, and Cr represents the expert authority coefficient. To ensure comparability between Ca and Cs, the raw score for each judgment basis was divided by 3 before calculating Ca. Ca was calculated as follows: $Ca =\frac{{{Ca}_{1}}/3+{{Ca}_{2}}/3+{{Ca}_{3}}/3+{{Ca}_{4}}/3}{4}$where ${Ca}_{1},{Ca}_{2},{Ca}_{3}$, and ${Ca}_{4}$ represent the raw scores for practical experience, theoretical analysis, literature evidence, and intuitive judgment, respectively. The expert authority coefficient was calculated as Cr = (Ca + Cs) / 2.
