# Supplementary material for: Developing a readiness evaluation framework for public hospital-led digital home nursing services in China: a Delphi-AHP study
Source: BMC Nurs. 2026 Aug 29;25:865. doi: 10.1186/s12912-026-05311-6 (PMC13589386; doi:10.1186/s12912-026-05311-6)
Supplement: Supplementary file 4 [file 12912_2026_5311_MOESM4_ESM.docx]

# Supplementary material 4

Supplementary Material 4 presents the consistency-testing and sensitivity-analysis results for the analytic hierarchy process (AHP) weighting procedure. Pairwise judgment matrices were constructed from the second-round Delphi importance scores using the predefined score-to-Saaty conversion rule described in the main manuscript. Table S11 reports the maximum eigenvalue (λmax), consistency index (CI), and consistency ratio (CR) for each judgment matrix. Among matrices for which CR was applicable, CR values ranged from 0.0088 to 0.0516, with all values below the prespecified threshold of 0.10. For two-indicator matrices, CI and CR were not applicable because pairwise comparison between two elements is inherently consistent. These results provide additional information on the internal consistency of the derived judgment matrices.

**Table S11.** Consistency test results of AHP judgement matrices.

| Comparison group | λ_max_ | C.I. | C.R. |
| --- | --- | --- | --- |
| 1 - 5 | 5.1947 | 0.0487 | 0.0435 |
| 1.1 - 1.2 | 2.0000 | - | - |
| 2.1 - 2.2 | 2.0000 | - | - |
| 3.1 - 3.3 | 3.0536 | 0.0268 | 0.0516 |
| 4.1 - 4.3 | 3.0536 | 0.0268 | 0.0516 |
| 5.1 - 5.4 | 4.0710 | 0.0237 | 0.0266 |
| 1.1.1-1.1.3 | 3.0536 | 0.0268 | 0.0516 |
| 1.2.1-1.2.3 | 3.0536 | 0.0268 | 0.0516 |
| 2.1.1-2.1.4 | 4.0710 | 0.0237 | 0.0266 |
| 2.2.1-2.2.3 | 3.0096 | 0.0046 | 0.0088 |
| 3.1.1-3.1.2 | 2.0000 | - | - |
| 3.2.1-3.2.3 | 3.0536 | 0.0268 | 0.0516 |
| 3.3.1-3.3.2 | 2.0000 | - | - |
| 4.1.1-4.1.3 | 3.0536 | 0.0268 | 0.0516 |
| 4.2.1-4.2.3 | 3.0183 | 0.0091 | 0.0176 |
| 4.3.1-4.3.3 | 3.0092 | 0.0046 | 0.0088 |
| 5.1.1-5.1.3 | 3.0536 | 0.0268 | 0.0516 |
| 5.2.1-5.2.3 | 3.0183 | 0.0091 | 0.0176 |
| 5.3.1-5.3.5 | 5.1433 | 0.0358 | 0.0320 |
| 5.4.1-5.4.3 | 3.0092 | 0.0046 | 0.0088 |

**Note:** λmax represents the maximum eigenvalue, CI the consistency index, and CR the consistency ratio. For two-indicator matrices, CI and CR were not applicable because the pairwise comparison is inherently consistent. Identical λmax, CI, and CR values across some comparison groups reflect structurally equivalent judgment matrices generated when the corresponding Delphi score differences fell within the same predefined Saaty-scale intervals. Each judgment matrix was nevertheless constructed independently from the second-round Delphi importance scores of its respective indicator group.

Sensitivity analyses were conducted to examine whether small changes in the predefined score-to-Saaty conversion boundaries materially affected the resulting indicator weights and rankings. Table S12 presents the detailed results for the second- and third-level indicators under the −0.05 and +0.05 perturbation scenarios. For each indicator, the baseline global weight and rank are shown alongside the recalculated values obtained under the two alternative specifications, together with the maximum absolute change in global weight.

At the second level, the overall priority structure remained stable across the alternative specifications. The highest-priority indicators retained their leading positions, while rank changes were mainly observed among indicators with relatively similar baseline weights. At the third level, most indicators showed only limited changes in global weight and rank. Several closely ranked indicators exchanged positions under the perturbed scenarios; however, the principal high-priority indicators remained stable. In particular, all 10 highest-ranked third-level indicators in the baseline analysis remained within the top 10 under both perturbation scenarios. Overall, the detailed results indicate that modest changes in the conversion boundaries affected some closely weighted indicators but did not substantially alter the main priority structure.

**Table S12.** Sensitivity analysis of second- and third-level indicator weights and rankings under alternative score-to-Saaty conversion boundaries.

| Indicator code | Baseline weight | Baseline link | Weight (-0.05) | Rank (-0.05) | Weight (+0.05) | Rank (+0.05) | Maximum absolute weight change |
| --- | --- | --- | --- | --- | --- | --- | --- |
| **Second-level** |  |  |  |  |  |  |  |
| 1.1 | 0.0937 | 4 | 0.0753 | 6 | 0.0937 | 4 | 0.0184 |
| 1.2 | 0.0468 | 9 | 0.0377 | 10 | 0.0468 | 9 | 0.0092 |
| 2.1 | 0.2152 | 1 | 0.2391 | 1 | 0.2152 | 1 | 0.0238 |
| 2.2 | 0.1076 | 3 | 0.1195 | 3 | 0.1076 | 3 | 0.0119 |
| 3.1 | 0.0342 | 11 | 0.0378 | 9 | 0.0343 | 11 | 0.0037 |
| 3.2 | 0.0814 | 5 | 0.0901 | 4 | 0.0814 | 5 | 0.0087 |
| 3.3 | 0.1292 | 2 | 0.1430 | 2 | 0.1292 | 2 | 0.0138 |
| 4.1 | 0.0632 | 7 | 0.0507 | 8 | 0.0632 | 7 | 0.0126 |
| 4.2 | 0.0266 | 12 | 0.0213 | 12 | 0.0266 | 12 | 0.0053 |
| 4.3 | 0.0167 | 14 | 0.0134 | 14 | 0.0167 | 14 | 0.0033 |
| 5.1 | 0.0770 | 6 | 0.0778 | 5 | 0.0770 | 6 | 0.0007 |
| 5.2 | 0.0198 | 13 | 0.0146 | 13 | 0.0198 | 13 | 0.0053 |
| 5.3 | 0.0343 | 10 | 0.0276 | 11 | 0.0343 | 10 | 0.0067 |
| 5.4 | 0.0543 | 8 | 0.0523 | 7 | 0.0543 | 8 | 0.0019 |
| **Third-level** |  |  |  |  |  |  |  |
| 1.1.1 | 0.0131 | 24 | 0.0105 | 26 | 0.0153 | 23 | 0.0026 |
| 1.1.2 | 0.0312 | 12 | 0.0250 | 15 | 0.0278 | 12 | 0.0061 |
| 1.1.3 | 0.0495 | 5 | 0.0398 | 8 | 0.0506 | 5 | 0.0097 |
| 1.2.1 | 0.0074 | 34 | 0.0059 | 34 | 0.0077 | 34 | 0.0014 |
| 1.2.2 | 0.0278 | 14 | 0.0224 | 18 | 0.0253 | 15 | 0.0055 |
| 1.2.3 | 0.0117 | 28 | 0.0094 | 28 | 0.0139 | 25 | 0.0023 |
| 2.1.1 | 0.0583 | 3 | 0.0624 | 4 | 0.0583 | 3 | 0.0041 |
| 2.1.2 | 0.0259 | 16 | 0.0284 | 11 | 0.0259 | 14 | 0.0025 |
| 2.1.3 | 0.0900 | 1 | 0.1079 | 1 | 0.0900 | 1 | 0.0179 |
| 2.1.4 | 0.0410 | 8 | 0.0404 | 7 | 0.0410 | 8 | 0.0006 |
| 2.2.1 | 0.0320 | 10 | 0.0355 | 10 | 0.0320 | 10 | 0.0035 |
| 2.2.2 | 0.0581 | 4 | 0.0645 | 3 | 0.0581 | 4 | 0.0064 |
| 2.2.3 | 0.0176 | 20 | 0.0195 | 19 | 0.0176 | 20 | 0.0019 |
| 3.1.1 | 0.0228 | 18 | 0.0252 | 14 | 0.0228 | 18 | 0.0024 |
| 3.1.2 | 0.0114 | 29 | 0.0126 | 24 | 0.0114 | 30 | 0.0012 |
| 3.2.1 | 0.0271 | 15 | 0.0242 | 16 | 0.0242 | 16 | 0.0029 |
| 3.2.2 | 0.0114 | 30 | 0.0106 | 25 | 0.0133 | 26 | 0.0019 |
| 3.2.3 | 0.0429 | 7 | 0.0553 | 5 | 0.0439 | 6 | 0.0124 |
| 3.3.1 | 0.0861 | 2 | 0.0953 | 2 | 0.0861 | 2 | 0.0092 |
| 3.3.2 | 0.0431 | 6 | 0.0477 | 6 | 0.0431 | 7 | 0.0046 |
| 4.1.1 | 0.0197 | 19 | 0.0150 | 22 | 0.0197 | 19 | 0.0046 |
| 4.1.2 | 0.0124 | 27 | 0.0083 | 31 | 0.0124 | 29 | 0.0041 |
| 4.1.3 | 0.0312 | 11 | 0.0273 | 13 | 0.0312 | 11 | 0.0039 |
| 4.2.1 | 0.0166 | 21 | 0.0133 | 23 | 0.0158 | 22 | 0.0033 |
| 4.2.2 | 0.0036 | 39 | 0.0029 | 40 | 0.0042 | 39 | 0.0007 |
| 4.2.3 | 0.0063 | 36 | 0.0051 | 36 | 0.0066 | 35 | 0.0013 |
| 4.3.1 | 0.0027 | 41 | 0.0022 | 41 | 0.0027 | 41 | 0.0005 |
| 4.3.2 | 0.0050 | 37 | 0.0040 | 37 | 0.0050 | 37 | 0.0010 |
| 4.3.3 | 0.0090 | 32 | 0.0072 | 33 | 0.0090 | 33 | 0.0018 |
| 5.1.1 | 0.0151 | 23 | 0.0152 | 21 | 0.0151 | 24 | 0.0001 |
| 5.1.2 | 0.0239 | 17 | 0.0242 | 17 | 0.0239 | 17 | 0.0002 |
| 5.1.3 | 0.0380 | 9 | 0.0384 | 9 | 0.0380 | 9 | 0.0004 |
| 5.2.1 | 0.0047 | 38 | 0.0035 | 38 | 0.0047 | 38 | 0.0013 |
| 5.2.2 | 0.0124 | 26 | 0.0091 | 29 | 0.0124 | 28 | 0.0033 |
| 5.2.3 | 0.0027 | 42 | 0.0020 | 42 | 0.0027 | 42 | 0.0007 |
| 5.3.1 | 0.0094 | 31 | 0.0075 | 32 | 0.0091 | 32 | 0.0019 |
| 5.3.2 | 0.0023 | 43 | 0.0016 | 43 | 0.0025 | 43 | 0.0007 |
| 5.3.3 | 0.0125 | 25 | 0.0099 | 27 | 0.0127 | 27 | 0.0026 |
| 5.3.4 | 0.0067 | 35 | 0.0057 | 35 | 0.0063 | 36 | 0.0011 |
| 5.3.5 | 0.0034 | 40 | 0.0030 | 39 | 0.0037 | 40 | 0.0004 |
| 5.4.1 | 0.0161 | 22 | 0.0155 | 20 | 0.0169 | 21 | 0.0008 |
| 5.4.2 | 0.0089 | 33 | 0.0086 | 30 | 0.0106 | 31 | 0.0018 |
| 5.4.3 | 0.0293 | 13 | 0.0282 | 12 | 0.0268 | 13 | 0.0025 |
