# Supplementary material for: Developing a readiness evaluation framework for public hospital-led digital home nursing services in China: a Delphi-AHP study
Source: BMC Nurs. 2026 Aug 29;25:865. doi: 10.1186/s12912-026-05311-6 (PMC13589386; doi:10.1186/s12912-026-05311-6)
Supplement: Supplementary file 5 [file 12912_2026_5311_MOESM5_ESM.docx]

**Expert Consultation Questionnaire on the Readiness Evaluation Framework for Public Hospital-Led Digital Home Nursing Services (Round 1)**

Dear Expert,

Thank you very much for taking the time to participate in this Delphi consultation.

With the rapid development of digital health technologies, professional nursing services are increasingly being extended from hospitals to patients’ homes. In China, Internet Plus Nursing Services have emerged as a policy-supported model of digital home nursing, in which registered nurses from medical institutions provide home-based nursing care through internet-based platforms. Since the National Health Commission launched the pilot program in 2019, this service model has gradually expanded from post-discharge continuing care to specialized nursing, chronic disease management, rehabilitation guidance, and other forms of home-based nursing care. As these services continue to develop, public hospitals are required to coordinate nursing workforce resources, digital platform infrastructure, payment arrangements, service quality, patient safety, and accountability to ensure the safe, sustainable, traceable, and standardized delivery of digital home nursing. However, existing studies have mainly focused on service models, patient demand and experience, nurses’ willingness to participate, and platform functionality, while limited attention has been paid to whether public hospitals possess the organizational and system readiness required to implement and sustain these services.

To address this gap, this study contextualizes Porter’s Five Forces model as an organizing framework for assessing the readiness of public hospital-led digital home nursing services. Based on a systematic review of the relevant literature, policy document analysis, and preliminary expert consultation, an initial indicator framework comprising **5** first-level domains, **16** second-level indicators, and **49** third-level indicators has been developed. To further assess the scientific validity, importance, feasibility, applicability, and practical relevance of the proposed indicators, we are conducting this Delphi consultation. We sincerely invite you to evaluate the indicators and provide suggestions regarding their revision, deletion, addition, or reclassification. Your professional judgment will provide an essential basis for refining and validating the final readiness evaluation framework.

This questionnaire consists of four sections. The first section collects basic information about the participating experts. The second section asks experts to rate the importance, feasibility, and applicability of each proposed indicator and to provide written comments where appropriate. The third section assesses experts’ familiarity with the research topic. The fourth section investigates the basis of expert judgment. Participation in this consultation is voluntary and anonymous. All information collected will be kept strictly confidential and used solely for academic research.

Once again, we sincerely appreciate your time, support, and valuable guidance. We look forward to receiving your expert feedback. We wish you good health and continued success in your professional endeavors.

Research Team

December 27, 2025

**Section 1 Expert Information**

**Instructions:**

The information collected in this section is intended solely for analyzing the characteristics of the expert panel participating in this Delphi consultation. All information will be kept strictly confidential and used exclusively for academic research. Please complete the questionnaire truthfully.

| Expert Information Form | | | | |
| --- | --- | --- | --- | --- |
| Age |  | | Gender |  |
| Institution |  | | Department |  |
| Highest Educational Qualification | | □ College Diploma □ Bachelor’s Degree □ Master’s Degree □ Doctoral Degree | | |
| Professional Background | | □ Clinical Nursing Professional □ Internet Healthcare Practitioner □ Healthcare Management Professional □ Health Services and Policy Researcher □ Other (please specify) _____________ | | |
| Professional Title | | □ Intermediate □ Associate Senior □ Senior | | |
| Years of Professional Experience | | □ 5-10 years □ 11-20 years □ 21-30 years □ More than 30 years | | |
| Have you participated in research or practice related to Internet Plus Nursing Services? | | □ Yes □ No | | |

**Section 2 Delphi Consultation Questionnaire**

**Instructions:**

1. Table 1 presents the preliminary readiness evaluation framework and the conceptual definitions of the proposed domains. Tables 2 and 3 present the initial indicator framework for expert evaluation. Table 4 and Table 5 are designed to assess your familiarity with the research topic and the basis of your judgments.

2. Please evaluate each proposed indicator in terms of importance, feasibility, and applicability using the following 5-point Likert scale: “ 5 = very important,” “ 4 = Improtant,” “ 3 = Moderately important,” “ 2 = Slightly important” and “ 1 = Not important”.

3. Please place a “ √ ” in the appropriate box for each rating. If you consider an indicator to be inappropriate, unclear, or unnecessary, please provide your comments in the Revision Suggestions column. If you believe that additional indicators should be included, please add them in the space provided and evaluate their importance, feasibility, and applicability.

**Table 1. Evaluation of the First-Level Readiness Domains**

| **First-level Domain** | **Description** | **Importance** | | | | | **Comments and Suggestions** |
| --- | --- | --- | --- | --- | --- | --- | --- |
| 1 Service Access and Institutional Requirements | Reflects the readiness of public hospitals to implement Internet Plus Nursing Services in terms of policy access, institutional qualifications, nurse practice requirements, and platform establishment. | □5 | □4 | □3 | □2 | □1 |  |
| 2 Workforce and Digital Platform Infrastructure | Reflects the readiness of nursing workforce resources and digital platform infrastructure, including workforce supply, professional competency, practice protection, platform functionality, system stability, and data security. | □5 | □4 | □3 | □2 | □1 |  |
| 3 Patient Adoption, Payment, and Value Recognition | Reflects patients’ acceptance and recognition of Internet Plus Nursing Services, including service affordability, medical insurance support, perceived value, convenience, safety, and trust. | □5 | □4 | □3 | □2 | □1 |  |
| 4 Substitute-Care Boundaries and Service Continuity | Reflects the influence of alternative care providers on Internet Plus Nursing Services and the clarity of professional care boundaries and continuity of care. | □5 | □4 | □3 | □2 | □1 |  |
| 5 Operational Quality, and Safety, and Accountability | Reflects the operational readiness of public hospitals in terms of service quality, safety, operational efficiency, accessibility, quality assurance, and accountability. | □5 | □4 | □3 | □2 | □1 |  |

**Table 2. Evaluation of the Second-Level Readiness Domains**

| **First-level Domain** | **Second-level Domain** | **Importance** | | | | | **Comments and Suggestions** |
| --- | --- | --- | --- | --- | --- | --- | --- |
| 1 Service Access and Institutional Requirements | 1.1 Policy and Regulatory Barriers to Internet Plus Nursing Services | □5 | □4 | □3 | □2 | □1 |  |
|  | 1.2 Platform Operation Costs for Internet Plus Nursing Services | □5 | □4 | □3 | □2 | □1 |  |
| 2 Workforce and Digital Platform Infrastructure | 2.1 Nurses’ Willingness to Participate in Internet Plus Nursing Services | □5 | □4 | □3 | □2 | □1 |  |
|  | 2.2 Diversity of Nursing Services Available Through Internet Plus Nursing Services | □5 | □4 | □3 | □2 | □1 |  |
| 3 Patient Adoption, Payment, and Value Recognition | 3.1 Price Sensitivity Toward Internet Plus Nursing Services | □5 | □4 | □3 | □2 | □1 |  |
|  | 3.2 Patients’ Awareness of the Relative Advantages of Internet Plus Nursing Services | □5 | □4 | □3 | □2 | □1 |  |
|  | 3.3 Medical Insurance Policy Support for Internet Plus Nursing Services | □5 | □4 | □3 | □2 | □1 |  |
|  | 3.4 Patients’ Demand for Extended Care Through Internet Plus Nursing Services | □5 | □4 | □3 | □2 | □1 |  |
| 4 Substitute-Care Boundaries and Service Continuity | 4.1 Accessibility of Traditional Nursing Services (Hospitals, Community Healthcare Facilities, and Home Care) | □5 | □4 | □3 | □2 | □1 |  |
|  | 4.2 Advantages of Internet Plus Nursing Services Provided by Non-Public Nursing Service Platforms | □5 | □4 | □3 | □2 | □1 |  |
|  | 4.3 Nursing Service Capacity of Elderly Care Institutions | □5 | □4 | □3 | □2 | □1 |  |
| 5 Operational Quality, and Safety, and Accountability | 5.1 Differentiation of Nursing Service Programs | □5 | □4 | □3 | □2 | □1 |  |
|  | 5.2 Nursing Service Charges and Cost Constraints | □5 | □4 | □3 | □2 | □1 |  |
|  | 5.3 Quality and Efficiency of Nursing Services | □5 | □4 | □3 | □2 | □1 |  |
|  | 5.4 Market Promotion and Service Coverage | □5 | □4 | □3 | □2 | □1 |  |
|  | 5.5 Platform Resource Coordination Capacity | □5 | □4 | □3 | □2 | □1 |  |

**Table 3. Evaluation of the third-Level Readiness Domains**

| **Second-level Domain** | **Third-level Domain** | **Importance** | | | | | **Comments and Suggestions** |
| --- | --- | --- | --- | --- | --- | --- | --- |
| 1.1 Policy and Regulatory Barriers to Internet Plus Nursing Services | 1.1.1 Policy and Regulatory Entry Requirements | □5 | □4 | □3 | □2 | □1 |  |
|  | 1.1.2 Nurse Qualification Requirements | □5 | □4 | □3 | □2 | □1 |  |
|  | 1.1.3 Policy Restrictions on Multi-site Nursing Practice | □5 | □4 | □3 | □2 | □1 |  |
| 1.2 Platform Operation Costs for Internet Plus Nursing Services | 1.2.1 Costs of Platform Development and Maintenance | □5 | □4 | □3 | □2 | □1 |  |
|  | 1.2.2 Costs of Equipment and Infrastructure Investment | □5 | □4 | □3 | □2 | □1 |  |
|  | 1.2.3 Costs of Marketing and Promotion | □5 | □4 | □3 | □2 | □1 |  |
|  | 1.2.4 Nursing Labor Costs | □5 | □4 | □3 | □2 | □1 |  |
| 2.1 Nurses’ Willingness to Participate in Internet Plus Nursing Services | 2.1.1 Nurses’ Willingness to Participate in Service Delivery | □5 | □4 | □3 | □2 | □1 |  |
|  | 2.1.2 Nurses’ Participation in Decisions on Service Income Distribution | □5 | □4 | □3 | □2 | □1 |  |
|  | 2.1.3 Nurses’ Pricing Autonomy for Different Service Items | □5 | □4 | □3 | □2 | □1 |  |
| 2.2 Diversity of Nursing Services Available Through Internet Plus Nursing Services | 2.2.1 Provision of Diverse Service Items for Different Patient Groups | □5 | □4 | □3 | □2 | □1 |  |
|  | 2.2.2 Incentive Policies for Part-time Nurses | □5 | □4 | □3 | □2 | □1 |  |
| 3.1 Price Sensitivity Toward Internet Plus Nursing Services | 3.1.1 Proportion of Out-of-Pocket Payments in Total Service Costs | □5 | □4 | □3 | □2 | □1 |  |
|  | 3.1.2 Price Advantage over In-person Nursing Services | □5 | □4 | □3 | □2 | □1 |  |
| 3.2 Patients’ Awareness of the Relative Advantages of Internet Plus Nursing Services | 3.2.1 Patients’ Perceived Convenience of the Service | □5 | □4 | □3 | □2 | □1 |  |
|  | 3.2.2 Patients’ Acceptance of the Standardization of Platform-Based Services | □5 | □4 | □3 | □2 | □1 |  |
|  | 3.2.3 Patients’ Perceived Safety of Nursing Services | □5 | □4 | □3 | □2 | □1 |  |
|  | 3.2.4 Patients’ Perception of the Comparability of Alternative Nursing Care Models | □5 | □4 | □3 | □2 | □1 |  |
| 3.3 Medical Insurance Policy Support for Internet Plus Nursing Services | 3.3.1 Number of Service Items Covered by Medical Insurance | □5 | □4 | □3 | □2 | □1 |  |
|  | 3.3.2 Proportion of Service Charges Reimbursed by Medical Insurance | □5 | □4 | □3 | □2 | □1 |  |
| 3.4 Patients’ Demand for Extended Care Through Internet Plus Nursing Services | 3.4.1 Patients’ Demand for Continuity with Inpatient or Outpatient Services | □5 | □4 | □3 | □2 | □1 |  |
|  | 3.4.2 Patients’ Demand for Home-Based Rehabilitation Nursing | □5 | □4 | □3 | □2 | □1 |  |
|  | 3.4.3 Patients’ Demand for Specialized Nursing Services | □5 | □4 | □3 | □2 | □1 |  |
| 4.1 Accessibility of Traditional Nursing Services (Hospitals, Community Healthcare Facilities, and Home Care) | 4.1.1 Convenience of In-person Hospital Nursing Services | □5 | □4 | □3 | □2 | □1 |  |
|  | 4.1.2 Feasibility of Home Hospital Bed Nursing Services | □5 | □4 | □3 | □2 | □1 |  |
|  | 4.1.3 Cost-effectiveness of Care Provided by Nursing Assistants | □5 | □4 | □3 | □2 | □1 |  |
| 4.2 Advantages of Internet Plus Nursing Services Provided by Non-Public Nursing Service Platforms | 4.2.1 Diversity of Nursing Service Items Provided by Non-public Platforms | □5 | □4 | □3 | □2 | □1 |  |
|  | 4.2.2 Capacity of Non-public Platforms to Provide Continuity with Inpatient or Outpatient Services | □5 | □4 | □3 | □2 | □1 |  |
|  | 4.2.3 Recognition of the Service Quality of Non-public Platforms | □5 | □4 | □3 | □2 | □1 |  |
| 4.3 Nursing Service Capacity of Elderly Care Institutions | 4.3.1 Feasibility of Community Nursing Services as a Substitute for Internet Plus Nursing Services | □5 | □4 | □3 | □2 | □1 |  |
|  | 4.3.2 Feasibility of Nursing Services Provided by Elderly Care Institutions as a Substitute for Internet Plus Nursing Services | □5 | □4 | □3 | □2 | □1 |  |
|  | 4.3.3 Capacity of Community and Institutional Nursing Services to Meet Patients’ Nursing Care Needs | □5 | □4 | □3 | □2 | □1 |  |
| 5.1 Differentiation of Nursing Service Programs | 5.1.1 Differentiation of Specialized Nursing Service Items | □5 | □4 | □3 | □2 | □1 |  |
|  | 5.1.2 Differences in the Coverage of Nursing Service Items | □5 | □4 | □3 | □2 | □1 |  |
|  | 5.1.3 Customized Nursing Service Packages Based on Patients’ Needs | □5 | □4 | □3 | □2 | □1 |  |
| 5.2 Nursing Service Charges and Cost Constraints | 5.2.1 Influence of Price Differences for Similar Nursing Services on Patients’ Choice | □5 | □4 | □3 | □2 | □1 |  |
|  | 5.2.2 Influence of Nursing Labor Costs on Service Pricing | □5 | □4 | □3 | □2 | □1 |  |
|  | 5.2.3 Influence of Platform Operating Costs on Service Pricing | □5 | □4 | □3 | □2 | □1 |  |
| 5.3 Quality and Efficiency of Nursing Services | 5.3.1 Timeliness of Internal Service Dispatch | □5 | □4 | □3 | □2 | □1 |  |
|  | 5.3.2 Smoothness of Nursing Service Delivery Processes | □5 | □4 | □3 | □2 | □1 |  |
|  | 5.3.3 Efficiency of Feedback and Problem Resolution | □5 | □4 | □3 | □2 | □1 |  |
|  | 5.3.4 Level of Personal Safety Protection for Nurses | □5 | □4 | □3 | □2 | □1 |  |
|  | 5.3.5 Level of Patient Safety Assurance | □5 | □4 | □3 | □2 | □1 |  |
| 5.4 Market Promotion and Service Coverage | 5.4.1 Promotion and Publicity of Internet Plus Nursing Services | □5 | □4 | □3 | □2 | □1 |  |
|  | 5.4.2 Convenience of Scheduling Nursing Services via Mobile Applications | □5 | □4 | □3 | □2 | □1 |  |
|  | 5.4.3 Accessibility of Internet Plus Nursing Services for Older Adults, People with Disabilities, and Individuals with Mobility Limitations | □5 | □4 | □3 | □2 | □1 |  |
| 5.5 Platform Resource Coordination Capacity | 5.5.1 Number of Full-time Nurses Available Through the Platform | □5 | □4 | □3 | □2 | □1 |  |
|  | 5.5.2 Integration of the Nursing Service Appointment App with Larger Platforms (e.g., Meituan and Alipay) | □5 | □4 | □3 | □2 | □1 |  |
|  | 5.5.3 Integration of Internet Plus Nursing Services with Medical Resources of Physical Hospitals | □5 | □4 | □3 | □2 | □1 |  |

**Section 3 Expert Self-Assessment Form**

**Instructions:**

1. Please select the basis for your judgments according to your actual situation. The primary sources of judgment include practical experience, theoretical analysis, domestic and international literature, and intuitive judgment. Please also indicate the degree of influence of each source (high, moderate, or low) by placing a “ √ ” in the appropriate box in Table 4.

2. Please indicate your level of familiarity with the research topic in Table 5. The response options are very familiar, familiar, moderately familiar, slightly familiar, and unfamiliar. Please place a “√” in the appropriate box.

**Table 4. Basis of Expert Judgment and Degree of Influence**

| Basis of Jugement | Degree of Influence | | |
| --- | --- | --- | --- |
|  | High | Moderate | Low |
| Practical Experience |  |  |  |
| Theoretical Analysis |  |  |  |
| Domestic and International Literature |  |  |  |
| Intuitive Judgment |  |  |  |

**Table 5. Expert Familiarity with the Research Topic**

| Level of Familiarity | Very Familiar | Familiar | Moderately Familiar | Slightly Familiar | Unfamiliar |
| --- | --- | --- | --- | --- | --- |
|  | 1.00 | 0.80 | 0.60 | 0.40 | 0.20 |
| Expert Self-Assessment |  |  |  |  |  |
