# Supplementary material for: Developing a readiness evaluation framework for public hospital-led digital home nursing services in China: a Delphi-AHP study
Source: BMC Nurs. 2026 Aug 29;25:865. doi: 10.1186/s12912-026-05311-6 (PMC13589386; doi:10.1186/s12912-026-05311-6)
Supplement: Supplementary file 6 [file 12912_2026_5311_MOESM6_ESM.docx]

**Expert Consultation Questionnaire on the Readiness Evaluation Framework for Public Hospital-Led Digital Home Nursing Services (Round 2)**

Dear Expert,

Thank you very much for your valuable comments and suggestions during the first round of the Delphi consultation. Based on the experts’ ratings and qualitative feedback obtained in the first round, the research team systematically revised the preliminary indicator framework according to the predefined screening criteria (mean importance score ≥ 3.5 and coefficient of variation ≤ 0.25), together with the experts’ recommendations for adding, deleting, modifying, and merging indicators. Following these revisions, the framework now consists of 5 first-level domains, 14 second-level indicators, and 41 third-level indicators, with several indicators merged or removed and new indicators added based on expert recommendations.

We are now conducting the second-round Delphi consultation to further achieve expert consensus and to validate and refine the revised indicator framework.

Thank you again for your continued support and valuable contribution to this study. We wish you good health and continued success in your professional endeavors.

Research Team

May 20, 2026

**Instructions for Completion**

**Instructions:**

1**. Table 1** presents the preliminary questionnaire domains and their corresponding conceptual definitions for your reference. **Tables 2** and **3** present the initial pool of indicators.

2. Please evaluate the importance and relevance of each proposed indicator. The importance of each indicator should be rated using the following 5-point Likert scale: “ 5 = very important,” “ 4 = Improtant,” “ 3 = Moderately important,” “ 2 = Slightly important” and “ 1 = Not important”. Please place a“√”in the appropriate box.

3. **Table 4** is designed to assess the basis of your judgments and the degree of influence of each source. Please select the basis for your judgments according to your actual situation and indicate its degree of influence. **Table 5** is designed to assess your level of familiarity with the consultation topic. Please select the appropriate level of familiarity and place a“√”in the corresponding box.

**Table 1. Evaluation of the First-Level Readiness Domains**

| **First-level Domain** | **Description** | **Importance** | | | | | **Comments and Suggestions** |
| --- | --- | --- | --- | --- | --- | --- | --- |
| 1 Service Access and Institutional Requirements | Reflects the readiness of public hospitals to implement Internet Plus Nursing Services in terms of policy access, institutional qualifications, nurse practice requirements, and platform establishment. | □5 | □4 | □3 | □2 | □1 |  |
| 2 Workforce and Digital Platform Infrastructure | Reflects the readiness of nursing workforce resources and digital platform infrastructure, including workforce supply, professional competency, practice protection, platform functionality, system stability, and data security. | □5 | □4 | □3 | □2 | □1 |  |
| 3 Patient Adoption, Payment, and Value Recognition | Reflects patients’ acceptance and recognition of Internet Plus Nursing Services, including service affordability, medical insurance support, perceived value, convenience, safety, and trust. | □5 | □4 | □3 | □2 | □1 |  |
| 4 Substitute-Care Boundaries and Service Continuity | Reflects the influence of alternative care providers on Internet Plus Nursing Services and the clarity of professional care boundaries and continuity of care. | □5 | □4 | □3 | □2 | □1 |  |
| 5 Operational Quality, and Safety, and Accountability | Reflects the operational readiness of public hospitals in terms of service quality, safety, operational efficiency, accessibility, quality assurance, and accountability. | □5 | □4 | □3 | □2 | □1 |  |

**Table 2. Evaluation of the Second-Level Readiness Domains**

| **First-level Domain** | **Second-level Domain** | **Importance** | | | | | **Comments and Suggestions** |
| --- | --- | --- | --- | --- | --- | --- | --- |
| 1 Service Access and Institutional Requirements | 1.1 Policy and Regulatory Barriers to Internet Plus Nursing Services | □5 | □4 | □3 | □2 | □1 |  |
|  | 1.2 Platform Operation Costs for Internet Plus Nursing Services | □5 | □4 | □3 | □2 | □1 |  |
| 2 Workforce and Digital Platform Infrastructure | 2.1 Nurses’ Willingness to Participate in Internet Plus Nursing Services | □5 | □4 | □3 | □2 | □1 |  |
|  | 2.2 Diversity of Nursing Services Available Through Internet Plus Nursing Services | □5 | □4 | □3 | □2 | □1 |  |
| 3 Patient Adoption, Payment, and Value Recognition | 3.1 Price Sensitivity Toward Internet Plus Nursing Services | □5 | □4 | □3 | □2 | □1 |  |
|  | 3.2 Patients’ Awareness of the Relative Advantages of Internet Plus Nursing Services | □5 | □4 | □3 | □2 | □1 |  |
|  | 3.3 Medical Insurance Policy Support for Internet Plus Nursing Services | □5 | □4 | □3 | □2 | □1 |  |
| 4 Substitute-Care Boundaries and Service Continuity | 4.1 Accessibility of Traditional Nursing Services | □5 | □4 | □3 | □2 | □1 |  |
|  | 4.2 Advantages of Internet Plus Nursing Services Provided by Non-public Nursing Service Platforms | □5 | □4 | □3 | □2 | □1 |  |
|  | 4.3 Nursing Service Capacity of Community and Elderly Care Institutions | □5 | □4 | □3 | □2 | □1 |  |
| 5 Operational Quality, and Safety, and Accountability | 5.1 Differentiation of Nursing Service Programs | □5 | □4 | □3 | □2 | □1 |  |
|  | 5.2 Nursing Service Charges and Cost Constraints | □5 | □4 | □3 | □2 | □1 |  |
|  | 5.3 Quality and Efficiency of Nursing Services | □5 | □4 | □3 | □2 | □1 |  |
|  | 5.4 Market Promotion and Service Coverage | □5 | □4 | □3 | □2 | □1 |  |

**Table 3. Evaluation of the third-Level Readiness Domains**

| **Second-level Domain** | **Third-level Domain** | **Importance** | | | | | **Comments and Suggestions** |
| --- | --- | --- | --- | --- | --- | --- | --- |
| 1.1 Policy and Regulatory Barriers to Internet Plus Nursing Services | 1.1.1 Policy and Regulatory Entry Requirements | □5 | □4 | □3 | □2 | □1 |  |
|  | 1.1.2 Nurse Qualification Requirements | □5 | □4 | □3 | □2 | □1 |  |
|  | 1.1.3 Policy Restrictions on Multi-site Nursing Practice | □5 | □4 | □3 | □2 | □1 |  |
| 1.2 Platform Operation Costs for Internet Plus Nursing Services | 1.2.1 Costs of Platform Development and Maintenance | □5 | □4 | □3 | □2 | □1 |  |
|  | 1.2.2 Costs of Equipment and Infrastructure Investment | □5 | □4 | □3 | □2 | □1 |  |
|  | 1.2.3 Costs of Marketing and User Acquisition | □5 | □4 | □3 | □2 | □1 |  |
| 2.1 Nurses’ Willingness to Participate in Internet Plus Nursing Services | 2.1.1 Nurses’ Willingness to Participate in Service Delivery | □5 | □4 | □3 | □2 | □1 |  |
|  | 2.1.2 Fairness of Nurses’ Income Distribution Mechanism | □5 | □4 | □3 | □2 | □1 |  |
|  | 2.1.3 Adequacy of the Supply of Specialized Nursing Personnel | □5 | □4 | □3 | □2 | □1 |  |
|  | 2.1.4 Completeness of the Occupational Risk Protection Mechanism for Nurses | □5 | □4 | □3 | □2 | □1 |  |
| 2.2 Diversity of Nursing Services Available Through Internet Plus Nursing Services | 2.2.1 Technical Platform Support for Nursing Services | □5 | □4 | □3 | □2 | □1 |  |
|  | 2.2.2 Stability of the Platform System and Level of Data Security | □5 | □4 | □3 | □2 | □1 |  |
|  | Dependence on External Technical Resources | □5 | □4 | □3 | □2 | □1 |  |
| 3.1 Price Sensitivity Toward Internet Plus Nursing Services | 3.1.1 Proportion of Out-of-Pocket Payments in Total Service Costs | □5 | □4 | □3 | □2 | □1 |  |
|  | 3.1.2 Price Advantage over In-person Nursing Services | □5 | □4 | □3 | □2 | □1 |  |
| 3.2 Patients’ Awareness of the Relative Advantages of Internet Plus Nursing Services | 3.2.1 Patients’ Perceived Value of Service Convenience and Accessibility | □5 | □4 | □3 | □2 | □1 |  |
|  | 3.2.2 Patients’ Acceptance of the Standardization, Safety, and Credibility of the Service | □5 | □4 | □3 | □2 | □1 |  |
|  | 3.2.3 Patients’ Perceived Relative Advantages over Other Nursing Care Models | □5 | □4 | □3 | □2 | □1 |  |
| 3.3 Medical Insurance Policy Support for Internet Plus Nursing Services | 3.3.1 Coverage of Internet Plus Nursing Service Items by Medical Insurance | □5 | □4 | □3 | □2 | □1 |  |
|  | 3.3.2 Level of Medical Insurance Reimbursement | □5 | □4 | □3 | □2 | □1 |  |
| 4.1 Accessibility of Traditional Nursing Services | 4.1.1 Accessibility of In-person Nursing Services Provided by Healthcare Institutions | □5 | □4 | □3 | □2 | □1 |  |
|  | 4.1.2 Availability of Home Hospital Beds and Related Home-Based Nursing Services | □5 | □4 | □3 | □2 | □1 |  |
|  | 4.1.3 Substitutability of Nursing Assistants or Other Non-professional Caregivers for Basic Nursing Care | □5 | □4 | □3 | □2 | □1 |  |
| 4.2 Advantages of Internet Plus Nursing Services Provided by Non-public Nursing Service Platformss | 4.2.1 Diversity of Nursing Service Items Provided by Non-public Platforms | □5 | □4 | □3 | □2 | □1 |  |
|  | 4.2.2 Capacity of Non-public Platforms to Provide Continuity with Inpatient or Outpatient Services | □5 | □4 | □3 | □2 | □1 |  |
|  | 4.2.3 Patients’ Recognition of the Service Quality of Non-public Platforms | □5 | □4 | □3 | □2 | □1 |  |
| 4.3 Substitute Capacity of Community and Elderly Care Services | 4.3.1 Feasibility of Community Nursing Services as a Substitute | □5 | □4 | □3 | □2 | □1 |  |
|  | 4.3.2 Feasibility of Nursing Services Provided by Elderly Care Institutions as a Substitute | □5 | □4 | □3 | □2 | □1 |  |
|  | 4.3.3 Feasibility of Emerging Nursing Care Models as a Substitute | □5 | □4 | □3 | □2 | □1 |  |
| 5.1 Differentiation of Nursing Service Programs | 5.1.1 Differentiation of Specialized Nursing Service Items | □5 | □4 | □3 | □2 | □1 |  |
|  | 5.1.2 Differentiation in the Coverage of Nursing Service Items | □5 | □4 | □3 | □2 | □1 |  |
|  | 5.1.3 Availability of Personalized Nursing Service Packages | □5 | □4 | □3 | □2 | □1 |  |
| 5.2 Nursing Service Charges and Cost Constraints | 5.2.1 Constraints of Nursing Labor Costs | □5 | □4 | □3 | □2 | □1 |  |
|  | 5.2.2 Constraints of Platform Operating Costs | □5 | □4 | □3 | □2 | □1 |  |
|  | 5.2.3 Reasonableness of Nursing Service Pricing | □5 | □4 | □3 | □2 | □1 |  |
| 5.3 Quality and Efficiency of Nursing Services | 5.3.1 Timeliness of Internal Service Dispatch | □5 | □4 | □3 | □2 | □1 |  |
|  | 5.3.2 Efficiency of Nursing Service Delivery | □5 | □4 | □3 | □2 | □1 |  |
|  | 5.3.3 Efficiency of Feedback and Problem Resolution | □5 | □4 | □3 | □2 | □1 |  |
|  | 5.3.4 Level of Personal Safety Protection for Nurses | □5 | □4 | □3 | □2 | □1 |  |
|  | 5.3.5 Level of Patient Safety Assurance | □5 | □4 | □3 | □2 | □1 |  |
| 5.4 Market Promotion and Service Coverage | 5.4.1 Service Promotion and Public Awareness | □5 | □4 | □3 | □2 | □1 |  |
|  | 5.4.2 Convenience of Mobile Appointment Scheduling | □5 | □4 | □3 | □2 | □1 |  |
|  | 5.4.3 Accessibility of Internet Plus Nursing Services for Older Adults, People with Disabilities, and Individuals with Mobility Limitations | □5 | □4 | □3 | □2 | □1 |  |

**Table 4. Basis of Expert Judgment and Degree of Influence**

| Basis of Jugement | Degree of Influence | | |
| --- | --- | --- | --- |
|  | High | Moderate | Low |
| Practical Experience |  |  |  |
| Theoretical Analysis |  |  |  |
| Domestic and International Literature |  |  |  |
| Intuitive Judgment |  |  |  |

**Table 5. Expert Familiarity with the Research Topic**

| Level of Familiarity | Very Familiar | Familiar | Moderately Familiar | Slightly Familiar | Unfamiliar |
| --- | --- | --- | --- | --- | --- |
|  | 1.00 | 0.80 | 0.60 | 0.40 | 0.20 |
| Expert Self-Assessment |  |  |  |  |  |
